# Inflammatory and glycolytic programs underpin a primed blood neutrophil state in patients with pneumonia

**DOI:** 10.1016/j.isci.2023.107181

**Published:** 2023-06-19

**Authors:** Alex R. Schuurman, Joe M. Butler, Erik H.A. Michels, Natasja A. Otto, Xanthe Brands, Bastiaan W. Haak, Fabrice Uhel, Augustijn M. Klarenbeek, Daniël R. Faber, Bauke V. Schomakers, Michel van Weeghel, Alex F. de Vos, Brendon P. Scicluna, Riekelt H. Houtkooper, W. Joost Wiersinga, Tom van der Poll

**Affiliations:** 1 Center for Experimental and Molecular Medicine (CEMM), Amsterdam University Medical Centers - Location AMC, University of Amsterdam, 1105 AZ Amsterdam, the Netherlands; 2 BovenIJ Hospital, Statenjachtstraat 1, 1034 CS Amsterdam, the Netherlands; 3 Laboratory Genetic Metabolic Diseases, Amsterdam UMC, University of Amsterdam, Departments of Clinical Chemistry and Pediatrics, Amsterdam Gastroenterology Endocrinology Metabolism, 1105 AZ Amsterdam, the Netherlands; 4 Core Facility Metabolomics, Amsterdam UMC, 1105 AZ Amsterdam, the Netherlands; 5 Department of Applied Biomedical Science, Faculty of Health Sciences, Mater Dei Hospital, University of Malta, Msida, Malta; 6 Amsterdam Gastroenterology Endocrinology and Metabolism Institute, 1105 AZ Amsterdam, the Netherlands; 7 Amsterdam Cardiovascular Sciences Institute, 1105 AZ Amsterdam, the Netherlands; 8 Division of Infectious Diseases, Amsterdam University Medical Centers - Location AMC, University of Amsterdam, 1105 AZ Amsterdam, the Netherlands

**Keywords:** Health sciences, Immunology, Medicine

## Abstract

Neutrophils are potent immune cells with key antimicrobial functions. Previous *in vitro* work has shown that neutrophil effector functions are mainly fueled by intracellular glycolysis. Little is known about the state of neutrophils still in the circulation in patients during infection. Here, we combined flow cytometry, stimulation assays, transcriptomics, and metabolomics to investigate the link between inflammatory and metabolic pathways in blood neutrophils of patients with community-acquired pneumonia. Patients’ neutrophils, relative to neutrophils from age- and sex- matched controls, showed increased degranulation upon *ex vivo* stimulation, and portrayed distinct upregulation of inflammatory transcriptional programs. This neutrophil phenotype was accompanied by a high-energy state with increased intracellular ATP content, and transcriptomic and metabolic upregulation of glycolysis and glycogenolysis. One month after hospital admission, these metabolic and transcriptomic changes were largely normalized. These data elucidate the molecular programs that underpin a balanced, yet primed state of blood neutrophils during pneumonia.

## Highlights

• The transcriptomic, metabolomic and functional profile of neutrophils in CAP
• These data provide *in vivo* verification that glycolysis fuels neutrophil activation
• Transcriptomic and metabolic changes largely normalize one month post-admission

## Graphical abstract

**Figure:**
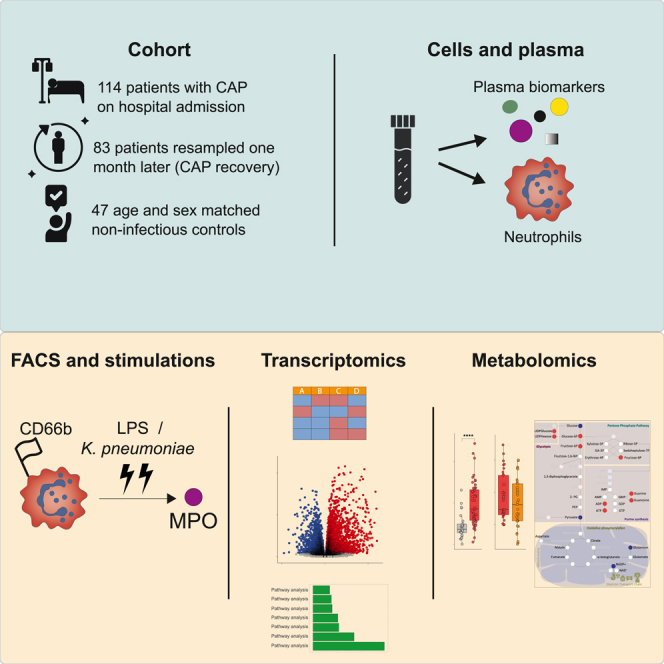

## Introduction

Neutrophils are quintessential antimicrobial immune cells.^1^^,^^2^ The importance of neutrophils in the host response against invading pathogens is illustrated by the numerous observations that neutrophil depletion results in higher susceptibility to infection, impaired microbial clearance and detrimental outcomes both in mice and humans.^2^^,^^3^^,^^4^ Neutrophils are not only key for their antimicrobial killing capacities, but also through their ability to shape the local inflammatory milieu through the production of proteinases, cytokines, chemokines, and other mediators. However, the inflammation that is entwined with neutrophil-mediated pathogen clearance can result in profound immunopathology: the balance between microbial killing and collateral tissue damage is delicate.^1^^,^^2^^,^^5^

The long-term consensus is that neutrophils fuel their effector functions through glycolysis.^6^^,^^7^ Recent *in vitro* work has nuanced this notion, showing that neutrophils are metabolically adaptive: as nutrient and oxygen levels at the site of infection are often low, neutrophils employ a variety of metabolic programs to maintain effector functionality.^8^^,^^9^ Dynamic glycogen stores can support glycolysis when glucose is scarce,^8^ and neutrophils can scavenge and metabolize extracellular proteins to create energy and granules.^9^ While the immunometabolism that facilitates neutrophil effector functions *in vitro* has thus been clarified, it remains uncertain how this translates to the *in vivo* state of neutrophils during infection. A recent study in patients with sepsis—a life-threatening condition that involves profound systemic immune dysregulation^10^—suggests that glycolysis is indeed important for neutrophil functionality in that setting, and that inhibition of glycolysis may lead to neutrophil immunosuppression.^11^ The blood neutrophil state prior to this stage of infection, preceding initiation of effector functions and before the onset of sepsis and sepsis-related interventions, remains unknown. It would be highly informative to understand the molecular profiles that dictate the state of circulating neutrophils in earlier stages of infection, as this would constitute an ideal time frame for neutrophil-modulating therapies seeking to control collateral tissue damage.

In this study, we sought to obtain insight into the transcriptional and metabolic programs that underpin the inflammatory state of blood neutrophils of patients with a relatively mild infection. To this end, we isolated blood neutrophils from non-critically ill patients with community-acquired pneumonia (CAP) at hospital admission and matched non-infectious controls, and assessed neutrophil activation status through flow cytometry and degranulation upon *ex vivo* stimulation, and performed untargeted transcriptomics and metabolomics to comprehensively profile the cells. Moreover, to determine whether observed changes were persistent or transient, we reanalyzed the majority of patients one month after hospital admission (CAP recovery).

## Results

### Patients and controls

**Figure 1:**
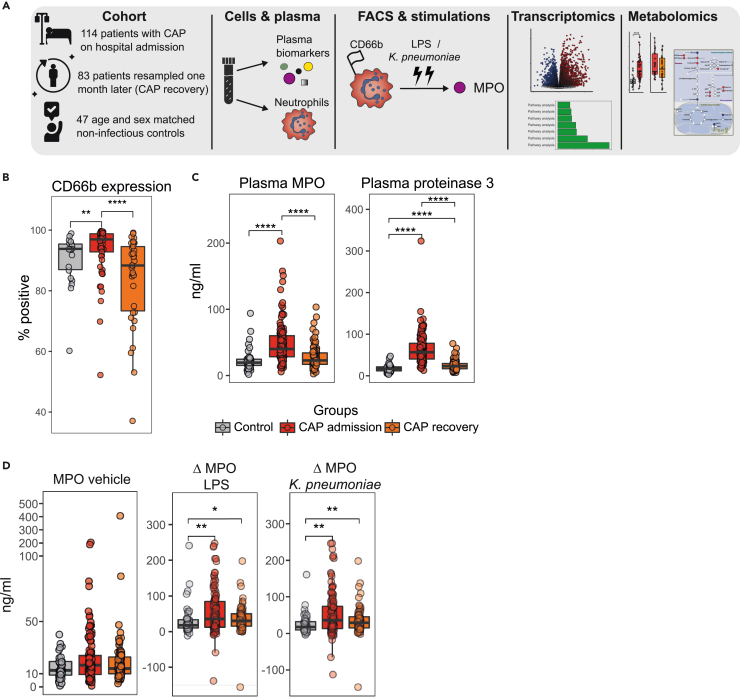
Blood neutrophils of patients with CAP are primed and pro-inflammatory (A) Study overview. (B) Percentage of CD66b positive neutrophils (22 control, 57 CAP admission, 35 CAP recovery). See Figure S1 for the gating strategy. (C) Plasma levels of MPO and proteinase 3 (47 control, 114 CAP admission, 88 CAP recovery). (D) MPO release of neutrophils after *ex vivo* stimulation (47 control, 114 CAP admission, 88 CAP recovery); delta (Δ) values represent the stimulated value (with LPS or *K. pneumoniae* respectively) minus the unstimulated (vehicle) value. Data are shown as boxplots with individual data points. ∗ p value < 0.05, ∗∗ p value < 0.01, ∗∗∗∗ p value < 0.0001 (by Wilcoxon rank-sum test).

We included 114 patients with CAP at hospital admission (CAP admission, Table 1). All patients were sampled within 24 h after presentation to the hospital. Patients were moderately ill considering disease severity scores and a low mortality (4.5% at day 28). We included 47 non-infectious controls, which were age and sex matched (controls: mean age 69.8 years [SD 8.3]; patients: mean age 67.5 years [SD 15.4], p = 0.227; controls: 55% male; patients: 55% male, p > 0.999). We resampled 83 (73%) patients one month (mean 33 days, SD 6 days) after hospital admission (CAP recovery). See Figure 1A for a graphical overview of the study.

**Table 1:** Baseline and clinical characteristics of the cohort

	Controls (N = 47)	CAP admission (N = 114)	p-value
**Demographics**			

Age	69.81 (8.31)	67.53 (15.37)	0.227
Sex, male	26 (55.3)	63 (55.3)	>0.999

**Comorbidities**			

Chronic obstructive pulmonary disease	4 (8.5)	35 (30.7)	0.002
Asthma	3 (6.4)	8 (7.0)	>0.999
Myocardial infarction	5 (10.6)	21 (18.4)	0.250
Stroke	1 (2.1)	13 (11.4)	0.068
Diabetes mellitus, type 2	4 (8.5)	28 (24.6)	0.028
Chronic kidney disease	3 (6.4)	14 (12.3)	0.399

**Routine lab results**			

Thrombocytes (x10^9^/L)		236.14 (111.49)	
Leukocytes (x10^9^/L)		12.00 [8.57, 15.93]	
Neutrophils (x10^9^/L)		9.80 [6.83, 13.48]	
Lymphocytes (x10^9^/L)		0.96 [0.61, 1.43]	
Neutrophil-to-lymphocyte ratio		10.45 [6.12, 15.69]	

**Disease severity**			

Pneumonia Severity Index		4 [3, 4]	
CURB-65		2 [1, 2]	
qSOFA		1 [0, 1]	

**Outcome**			

Hospital length of stay (days)		7.8 (10.5)	
28-day mortality		5 (4.5)	

CURB-65 = Confusion Urea Respiratory rate Blood pressure age 65; qSOFA = quick sequential organ failure assessment score; LOS = length of stay. Continuous data are presented as mean (standard deviation) or median [IQR], and compared using a two-sided ANOVA or two-sided Kruskal-Wallis test, respectively. Categorical data are presented as count (percentage) and compared using Fisher’s exact test.

### Blood neutrophils in patients with CAP are primed and pro-inflammatory

We first assessed neutrophil CD66b expression—a marker for neutrophil priming^12^ and specific/secondary granule content^13^—by flow cytometry (see Figure S1 for the gating strategy). The proportion of CD66b positive neutrophils was significantly increased in patients with CAP at admission, suggesting a primed state (Figure 1B). The plasma levels of the neutrophil degranulation products myeloperoxidase (MPO) and proteinase 3 were strongly elevated in patients with CAP (Figure 1C), as were the plasma levels of neutrophil-activating cytokines interleukin (IL)-6, IL-8 and granulocyte colony-stimulating factor (G-CSF), and markers of systemic inflammation (C-reactive protein and ferritin) (Figure S2). Neutrophil CD66b expression and plasma MPO levels had normalized one month after hospital admission, while the plasma levels of proteinase 3 remained modestly elevated compared to controls. Next, we assessed the functional phenotype of blood neutrophils by *ex vivo* stimulation (2 h) of isolated cells with lipopolysaccharide (LPS) or heat-killed *Klebsiella pneumoniae*, one of the main causative agents of bacterial pneumonia in hospitalized patients.^14^ While we observed no differences in the unstimulated (medium) condition (reflecting spontaneous degranulation), neutrophils from patients admitted for CAP clearly released more MPO upon stimulation than controls, indicating an activated, pro-inflammatory phenotype (Figure 1D). Interestingly, increased MPO release upon stimulation was partially maintained after CAP recovery, indicating persistent hyperresponsiveness to Toll-like receptor (TLR)-stimulation one month after hospital admission.

### Metabolic and inflammatory programs underpin a primed neutrophil state

**Figure 2:**
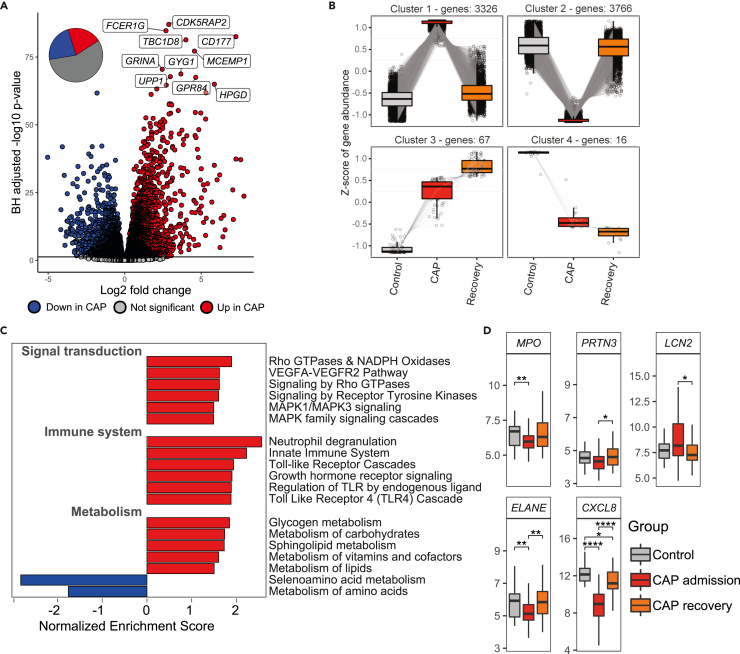
Metabolic and inflammatory programs underpin a primed neutrophil state (A) Volcano plot showing the differentially expressed genes (DEGs) in neutrophils from 64 patients with CAP (admission sample), and 29 controls. Each dot represents a gene. The X axis denotes the log2 fold change between groups, while the Y axis shows the Benjamini-Hochberg adjusted -log10 p value. The top 10 DEGs are annotated. The piechart represents the whole transcriptome and indicates what percentage of genes was upregulated, downregulated, or unchanged. (B) Boxplots showing four clusters of correlated genes that portray specific trends between controls, CAP admission and CAP recovery (29 controls, 64 CAP admission, 34 CAP recovery), as described in the Methods. Only genes that were significantly different between CAP admission and controls were used in this analysis. (C) Results of the Reactome pathway analysis of all DEGs between patients with CAP (admission sample) and controls. Depicted are the significantly different “child” pathways in Reactome relating to the following “mother” pathways: “signal transduction,” “immune system,” and “metabolism;” for “immune system” only the top six pathways are depicted, see Table S2 for data on all significant child pathways in this mother pathway. All significant mother pathways are shown in Figure S3A. (D) Boxplots showing the expression of selected genes between the groups. Differences were tested with a Wilcoxon rank-sum test. ∗ p value < 0.05, ∗∗ p value < 0.01, ∗∗∗∗ p value < 0.0001.

To explore the transcriptional profile that underpins this activated and primed neutrophil state, we performed RNA-sequencing of isolated neutrophils in a subset of the cohort. A direct comparison between patients with CAP at admission and controls indicated a profound shift in the neutrophil transcriptional landscape, with 3398 (20.6%) significantly upregulated, and 3779 (22.9%) significantly downregulated genes in patients (Figure 2A). Notably, direct comparison between CAP recovery samples and controls yielded no—after correction for multiple testing—significant differentially expressed genes (DEGs). To further assess dynamic transcriptomic changes from health to CAP to CAP recovery, we calculated clusters of genes with the same trajectory between the groups as described in the Methods section. This analysis indicated that the vast majority of DEGs at CAP admission returned to control levels after CAP recovery (Figure 2B). Two small clusters of genes were either persistently downregulated or upregulated, but overrepresentation analysis identified no specific Reactome^15^ pathways within these clusters (Table S1). To aid interpretation of the DEGs between patients with CAP and controls, we performed gene set enrichment analysis.^16^ This approach identified signal transduction, immune system, and metabolism to be the most significantly enriched “mother” pathways (pathways at the top of the Reactome pathway hierarchy, Figure S3A). For these three primary pathways, we zoomed in on their respective “child” pathways, which revealed enrichment of several signaling protein families (rho GTPase, tyrosine kinase, MAPK) as part of signal transduction (Figure 2C, top 6 pathways are plotted for “immune system,” see Table S2 for all pathways of “immune system”). Among the most enriched immune system related pathways were neutrophil degranulation, TLR cascades, and interleukin signaling. Figure S3B shows the top 10% upregulated and top 10% downregulated genes of the 299 significantly altered genes (70 down, 229 up) in neutrophil degranulation, illustrating how this profile was normalized after CAP recovery (see Table S3 for information on all DEGs in this pathway). Targeted analysis of genes coding for degranulation products showed reduced expression of *MPO*, *ELANE* (elastase), and *CXCL8* (IL-8) in neutrophils of patients with CAP at admission, and unaltered *PRTN3* (proteinase 3), and *LCN2* (NGAL) expression (Figure 2D), suggesting that the exaggerated release of MPO by neutrophils of patients with CAP after *ex vivo* stimulation was not caused by ongoing MPO synthesis. With regard to metabolism we observed a transcriptional increase of genes implicated in glycogen, carbohydrate, and lipid metabolism in neutrophils from CAP patients, while amino acid metabolism was downregulated (Figure 2C).

### Weighted gene co-expression network analysis links inflammatory and glycolytic pathways

**Figure 3:**
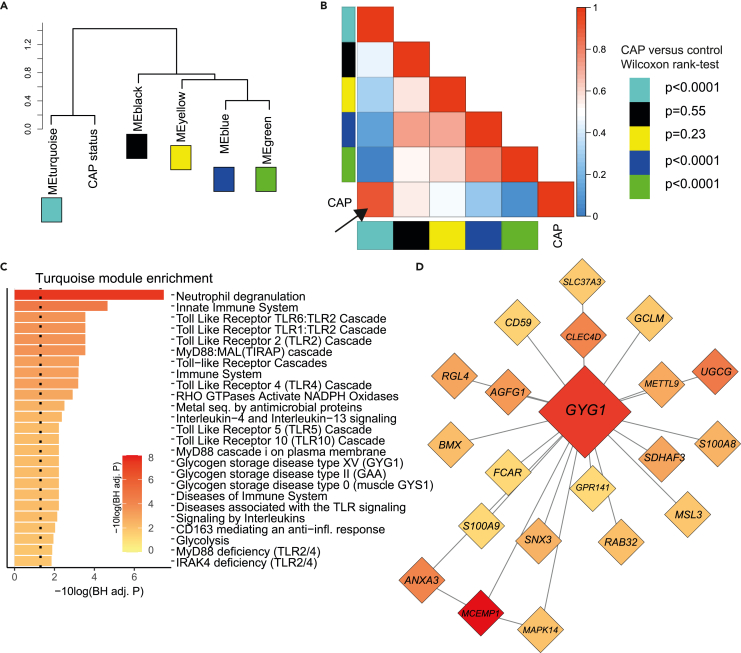
Weighted gene co-expression network analysis indicates links between inflammatory and glycolytic pathways (A) Hierarchical clustering of module eigengenes and CAP (see Supplemental Methods for details). The cluster tree shows that CAP is most related to the turquoise cluster. ME = module eigengene. (B) Heatmap of the adjacencies in the eigengene network showing the module-module and module-trait (CAP) relationships. Modules are labeled by their corresponding color. In the heatmap red colors indicate high adjacency (positive correlation) and blue colors indicate low adjacency (negative correlation). p-values on the right of the plot denote the results of comparing the module scores between patients with CAP at admission and controls with a Wilcoxon rank-sum test. (C) Results of Reactome over representation analysis, showing which pathways are significantly enriched within the turquoise module. (D) Network plot showing the top 20 most important genes in the turquoise model, as well as the hub gene *GYG1*. The color indicates the expression difference between patients with CAP and controls, wherein red means higher levels in patients with CAP.

To substantiate the comparative pathway analyses, we performed weighted gene co-expression network analysis (WGCNA, an unbiased method to find modules of highly related genes^17^^,^^18^^,^^19^). Eight modules of co-expressed genes were identified, of which five remained after merging of similar modules (see Supplemental Methods and Figures S4–S6 for technical results). Hierarchical clustering indicated that the turquoise module was most closely linked to CAP (Figure 3A). Indeed, turquoise was the only module with a positive correlation (rho = 0.80, p < 0.0001) to CAP, and the module eigengene scores, used to compare module relationships between groups, were very significantly different between CAP and controls (Wilcoxon rank-test p < 0.0001, Figure 3B). The genes entailed within this turquoise module were mainly representative for transcriptional programs of neutrophil degranulation, innate immune system, and TLR-cascades (Figure 3C), which is fully in line with our gene set enrichment analysis (Figure 2C). Interestingly, glycogen and glycolysis-related pathways were also represented in the turquoise module, suggesting a link between metabolic and immune-related pathways. Indeed, the hub gene of this module was *GYG1*—coding for glycogenin 1—which plays a role in glycogen storage (Figure 3D).

### Interplay between metabolic pathways, immune activation, and systemic inflammation

**Figure 4:**
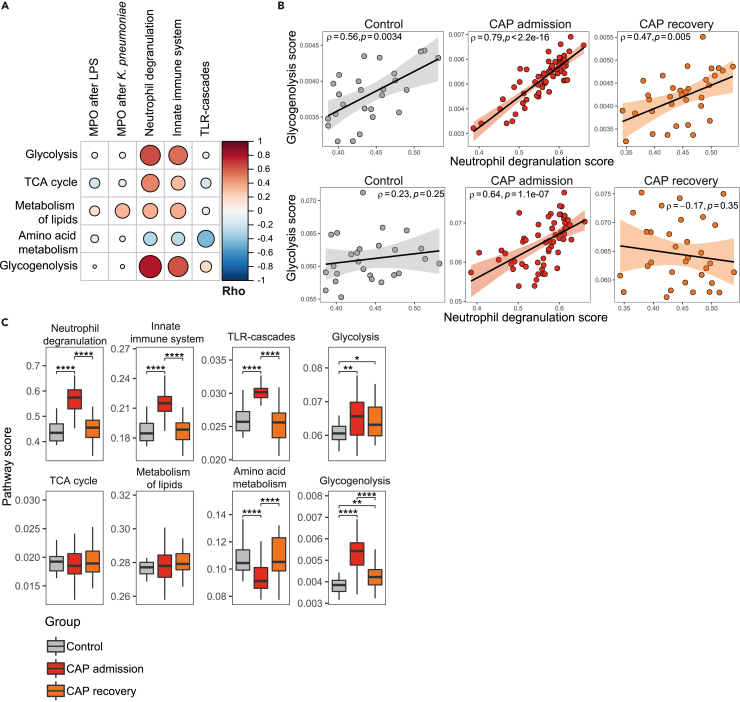
Interplay between neutrophil metabolic pathways, neutrophil activation, and systemic inflammation (A) Correlation matrix depicting the association between metabolic pathway scores, and *ex vivo* MPO release after stimulation and immune-related pathway scores. Pathway scores (based on pathways in Reactome^15^^,^^16^) were calculated as described in the Methods. The color and size indicate the direction and strength of the correlation, quantified in Spearman’s rho. AA = amino acid, TCA = tricarboxylic acid. (B) Scatterplots showing the correlation between the neutrophil degranulation score, and the glycogenolysis and glycolysis score, respectively. The scatterplots and correlations are split between the three groups (29 controls, 64 CAP admission, 34 CAP recovery). The correlation is quantified with Spearman’s rho. (C) Boxplots showing the pathway scores between the three groups. Differences were tested with a Wilcoxon rank-sum test. ∗ p value < 0.05, ∗∗ p value < 0.01, ∗∗∗ p value < 0.001, ∗∗∗∗ p value < 0.0001.

We next sought to assess how metabolic programs related to the *ex vivo* MPO release and the induction of immune-related pathways in neutrophils. We calculated individual pathway scores per subject for the Reactome pathways neutrophil degranulation, innate immune system, TLR-cascades, and the major energy pathways glycolysis, the tricarboxylic acid (TCA) cycle, metabolism of lipids, amino acid metabolism, and glycogenolysis as described in the Methods. In line with the paradigm that glucose metabolism is important for neutrophil effector functions,^6^^,^^8^ glycolysis (rho 0.64, p < 0.0001) and glycogenolysis (rho 0.79, p < 0.0001) scores were strongly positively correlated with neutrophil degranulation in CAP patients on admission, whereas amino acid metabolism showed a negative correlation (Figure 4A). MPO release appeared not connected to transcriptional pathway activity, again suggesting that the MPO release capacity of circulating neutrophils is not shaped by the cellular transcriptome. Interestingly, the glycolysis-neutrophil degranulation correlation was only present in patients with CAP, particularly at admission, possibly reflecting a disease-effect, whereas the glycogenolysis-neutrophil degranulation relationship was stable in all groups (Figure 4B). Correlation of the individual pathway scores to plasma biomarkers revealed that IL-6 (rho = 0.62, p < 0.001) and G-CSF (rho = 0.52, p < 0.001) levels were most strongly related to upregulation of neutrophil degranulation programs, suggesting a role for the inflammatory milieu of the circulation in shaping the neutrophil transcriptome (see Figure S7 for all correlations). One month after hospital admission most transcriptional pathway activity was again similar to levels in controls, although glycogenolysis and glycolysis remained partially elevated (Figure 4C).

### Untargeted metabolomics confirms transcriptional patterns

**Figure 5:**
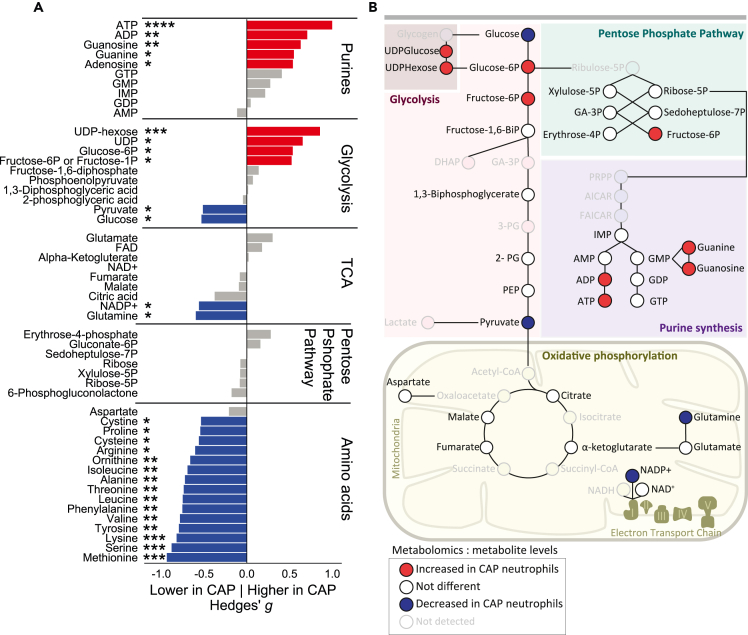
Untargeted metabolomics of neutrophils confirms their transcriptional patterns (A) Stacked barplots showing the effect size and significance for metabolites in purine synthesis, glycolysis, the TCA-cycle, the pentose phosphate pathway, and amino acids (26 control, 50 CAP admission). The X axis denotes the Hedges’ g effect size between the patients with CAP at hospital admission, and controls. The stars indicate the significance per metabolite calculated with Student’s *t* test on Box-Cox transformed values. ∗ p value < 0.05, ∗∗ p value < 0.01, ∗∗∗ p value < 0.001, ∗∗∗∗ p value < 0.0001. (B) Graphic overview of all pathways in panel A except for amino acids. The color of the node indicates whether the metabolite was significantly higher (red) or lower (blue) in neutrophils of patients with CAP when compared to controls, or not significantly different (white).

Our transcriptomic data suggested that glycolysis and glycogenolysis are important for the primed, poised state of circulating neutrophils in patients with CAP, and in addition indicated that amino acid metabolism was downregulated. To confirm these findings on a metabolite level, we performed untargeted metabolomics in lysates of purified neutrophils. A direct comparison between the neutrophil metabolomes of patients with CAP and controls showed substantial differences across several metabolic domains. ATP was the most significantly increased metabolite in patients’ neutrophils (indicative of a high-energy state, p = 4.88e-08), together with an increase of other purines (ADP, UDP, guanosine) (Figure 5A, see Figure S8 for a volcano plot). Most annotated glycolytic metabolites were significantly different, with increased glycogenolysis-related metabolites (UDPs) and glycolytic intermediates (glucose-6-phosphate and fructose-6-phosphate), but lower levels of glucose and pyruvate. Low pyruvate levels may correspond to decreased TCA activity, as we observed little TCA-metabolite differences between groups and significantly decreased levels of NADP^+^ and glutamine in patients. Amino acid levels were strikingly decreased (all but aspartate) in patients’ neutrophils, which confirms our transcriptomic pathway analyses on a metabolite level. Notably, CAP recovery metabolomic samples were again similar to controls (with only NADP^+^ (p = 0.042) and erythrose-4-phosphate (p = 0.044) significantly lower in patients), indicating that the neutrophil metabolome normalizes one month after hospital admission for CAP. Figure 5B illustrates these metabolic pathways (except for amino acids), highlighting the differential glycogenolysis-glycolysis pathway, high-energy state and increased purine synthesis in neutrophils of patients with CAP.

### Sub-analysis of the most severe cases

To explore the recovery of neutrophil profiles in patients with high disease severity, we separately analyzed patients with a Pneumonia Severity Index class of 4 (PSI score 91–130) or higher at hospital admission. We found a similar, partially maintained *ex vivo* hyperresponsiveness to TLR-stimulation of neutrophils one month after hospital admission (Figure S9A). Interestingly, while transcriptomic comparison of all CAP recovery samples to controls yielded no significantly expressed genes, we found that neutrophil transcriptomes of severe cases at one month after hospital admission did differ from controls (2 downregulated genes, 93 upregulated genes, Table S4, Figure S9B). Functional implications remain unclear; however, as pathway analysis did not identify any enriched, annotated pathways within these DEGs. Finally, metabolomic comparisons indicated that—similar to the primary analyses—the neutrophil metabolome of severe CAP cases was normalized at CAP recovery, as only NADP+ (p = 0.036) was significantly lower in patients when compared to controls. Together, these analyses suggest that neutrophil recovery is mostly similar for severe CAP cases, although we did observe persistent transcriptomic changes one month after hospital admission in this group.

## Discussion

Several studies have documented the importance of glycolysis for neutrophil effector functions.^6^^,^^8^^,^^11^^,^^20^ Importantly, however, this knowledge is mainly derived from *in vitro* experiments with purified neutrophils^6^^,^^8^^,^^20^ and indirect evidence from patients with full-blown sepsis suffering from a pronounced distortion from normal homeostasis at multiple levels.^11^ The state of neutrophils still in the circulation of patients with relatively mild infection *in vivo*, prior to full activation, sepsis, or tissue infiltration, remains poorly characterized. We here specifically chose to study pneumonia patients, considering the indisputable role of neutrophils in host defense during lower respiratory tract infection—associated with priming in the circulation with subsequent migration to the airways^14^^,^^21^—and its high disease burden around the globe.^22^^,^^23^ We used an integral approach, combining flow cytometry, stimulation assays, transcriptomics, and metabolomics, to obtain insight into the metabolic pathways contributing to the primed and pro-inflammatory state of blood neutrophils in patients with non-severe CAP.

We found a primed, pro-inflammatory phenotype of neutrophils in CAP patients at hospital admission relative to age- and sex-matched control subjects. Our main findings indicate that this phenotype was underpinned by a high intracellular energy state, associated with upregulation of glycolysis and glycogenolysis at both the transcriptomic and metabolomic level. These results provide the first verification in patients with a relative mild infection *in vivo* of the paradigm that glycolysis fuels neutrophil activation. Our data are in line with relatively recent work on the importance of glycogenolysis for neutrophils in different settings; for instance, the glycogen-glucose axis could maintain glycolysis in neutrophils under a hypoglycemic, hypoxic, and LPS-stimulated condition *ex vivo*.^8^ Our data in patients’ neutrophils corroborates this to some extent: we find transcriptional upregulation of glycogenolysis, and increased levels of glycogenolysis-intermediates UDP-glucose, UDP-hexose and glucose-6-phosphate. The setting is different, however, as we analyzed the cellular state as it would be in the circulation, ergo a normo-glycemic condition. This may explain why we find clear upregulation of glycolysis, which correlates with transcriptional pro-inflammatory responses. These data tie in with another study, which showed that circulating neutrophil functionality during sepsis is dependent on glycolysis.^11^ Our data expand on these findings by showing that this link already exists during “earlier” stages of the syndrome, and is not limited to sepsis. Notably, our group previously found that intracellular pyruvate levels in monocytes were correlated with cytokine production upon stimulation with LPS.^24^ We did not find similar correlations in neutrophils, nor for other metabolite levels, indicating a disconnect between the cellular metabolome and MPO release in neutrophils. Furthermore, we observed a striking decrease in amino acid metabolism, both on a transcriptomic and a metabolomic level. It was recently shown that in low-nutrient and hypoxic settings, murine pulmonary neutrophils scavenge and catabolize extracellular proteins to compensate for the decreased glycolysis capacity.^9^ Possibly circulating neutrophils are able to strongly decrease intracellular amino acid levels and downregulate amino acid metabolism, relying on the relatively high oxygen and nutrient levels in the blood. While glycolysis remains a key for effector functions, these data together point toward the metabolic flexibility of neutrophils in response to nutrient availability of the microenvironment.

Neutrophils of patients with CAP at hospital admission had a primed, yet balanced phenotype. Indeed, we observed distinct upregulation of inflammatory and signaling transcriptional programs, and increased degranulation *ex vivo,* but the latter only upon stimulation: MPO release at baseline was unchanged. Neutrophil granule formation and distribution is thought to be determined during granulopoiesis,^13^ and—depending on the timing of sampling—mRNA levels do not necessarily correlate with granule protein content.^25^ This was reflected in our data: while we observed an overall activated neutrophil phenotype in patients with CAP, expression of most granule protein mRNAs was decreased. Moreover, MPO release upon *ex vivo* stimulation was not clearly correlated with transcriptomic programs, suggesting a disconnect between granule release and transcriptomic regulation in circulating neutrophils.

Follow-up and reanalysis of patients with CAP revealed that the observed metabolic and pro-inflammatory transcriptomic features in neutrophils at hospital admission were largely normalized one month later. This suggests that the majority of observed changes were indeed attributable to the episode of pneumonia, rather than to baseline differences between patients and controls, and that the primed neutrophil phenotype is transient. Notably, the exaggerated MPO release upon stimulation was partially maintained after CAP recovery, suggesting persistent hyperresponsiveness of circulating neutrophils. Also, transcriptomic activity in the glycolysis and glycogenolysis pathways remained elevated in CAP recovery samples compared to controls. Considering the relatively short half-life of neutrophils in the circulation, these results suggest a partially altered granulopoiesis in the weeks following an acute episode of pneumonia.

Together, these data provide insight in the transcriptional and metabolic programs that underpin a primed, inflammatory blood neutrophil state in patients with CAP at hospital admission. This study provides the first *in vivo* verification of the paradigm that glycolysis fuels blood neutrophil activation in patients with a relatively mild infection, thereby linking previous experimental work to a relevant clinical setting. These results may inform immune-modulatory strategies seeking to dampen neutrophil-mediated inflammation in a relatively early stage of infection.

### Limitations of the study

Our study has strengths and limitations. Patients with pneumonia on the ward represent a large and relevant medical population, and variance introduced by invasive and immunomodulatory therapies remains limited (especially when compared to intensive care cohorts). The combined use of untargeted transcriptomics and metabolomics permitted assessment of metabolic pathways at multiple levels, which makes interpretation more robust. Follow-up of patients enabled separation of transient and enduring changes, and helped to determine whether observed features can be attributed to the episode of pneumonia. An inherent limitation of our approach is that—while we focused on ward patients—there is variance in disease severity between patients, which could influence our between-group and longitudinal comparisons. For example, we only observed persistent transcriptional changes in the most severely ill patients. Furthermore, as our main goal was to profile the neutrophil state in the circulation of patients, we included only limited *ex vivo* functional read-outs: addition of multiple cytokine measurements, or for instance phagocytosis assays, would have been preferable. Also, while our metabolomic panel was fairly extensive, NADH and lactate were lacking, which is a limitation when assessing glycolysis. In this study we focused on circulating neutrophils; although logistically and ethically challenging, future work could seek to expand these analyses to the bone marrow and/or airways of patients on the ward, to assess the transcriptional and metabolic dynamics of neutrophils as they transition between compartments. Furthermore, fluxomics would be a valuable approach to assess the dynamics of the metabolic state.^26^

## STAR★Methods

### Key resources table

**Table:** 

REAGENT or RESOURCE	SOURCE	IDENTIFIER
**Antibodies**		

anti-CD14-APC	BD Biosciences	Cat# 561383
anti-CD3-PECy7	BD Biosciences	Cat#563423
anti-CD66-FITC	BD Biosciences	Cat#551479

**Bacterial strains and products**		

Klebsiella pneumoniae	ATCC	ATCC43816
LPS; Escherichia coli	Invivogen, Toulouse	0111:B4 Ultrapure

**Critical commercial assays**		

AllPrep DNA/RNA mini kit	Qiagen, Germany	Cat#80004

### Resource availability

#### Lead contact

A.R. Schuurman, MD, contact information: a.r.schuurman@amsterdamumc.nl.

#### Materials availability

This study did not generate new unique reagents.

### Experimental model and study participant details

The ELDER-BIOME study was performed in Amsterdam UMC, location Academic Medical Center (AMC), and the BovenIJ hospital in the Netherlands from October 2016 - June 2017 and October 2017 - June 2018 (clinicaltrials.gov identifier NCT02928367).^27^^,^^28^ Written informed consent was obtained from all eligible participants or their legal representatives. The study protocol was approved by the local institutional review boards (reference: NL57847.018.16) and conducted according to the declaration of Helsinki. Age- and sex matched subjects without acute infection who presented at the outpatient clinic of Amsterdam UMC, location AMC, were included as controls, after written informed consent was obtained. Patients older than 18 years admitted to the hospital were screened by trained research physicians. Patients were included if they were admitted with a clinical suspicion of an acute infection of the respiratory tract, defined as the presence of at least two diagnostic clinical criteria (new cough or sputum production, dyspnea, tachypnoea, hypoxemia, abnormal lung examination, documented fever or hypothermia, leukocytosis or C-reactive protein levels >3 times above the upper limit), combined with an evident new or progressive infiltrate, consolidation, cavitation, or pleural effusion on chest X-ray or computed tomography scan. Patients with the clinical suspicion of an aspiration pneumonia, an obvious non-respiratory source of infection, or patients who had recently been hospitalized (for >48 h in the previous 2 weeks), or who resided in long-term care facilities, were not considered to have a working diagnosis of CAP.

### Method details

#### Host response biomarker measurements

EDTA anticoagulated plasma was obtained within 24 h after hospital admission. MPO, proteinase 3, IL-6, IL-8, G-CSF, C-reactive protein and ferritin were measured using Luminex multiplex assay (R&D, Minneapolis, MN) and BioPlex 200 (BioRad, Hercules, CA) following the manufacturer’s instructions. Measurements below the limit of quantification were imputed as half the lower limit of quantification. Luminex measurements above the upper limit of quantification were set to the upper limit of quantification.

#### Neutrophil isolation

Heparin anticoagulated blood was collected within 24 h of hospital admission, and one month thereafter. Polymorphonuclear cells were isolated from the lowest fraction of Ficoll-Paque separated heparin tubes, and erythrocytes were lysed with erythrocyte lysis buffer (Sigma-Aldrich, Saint Louis, Missouri). Cell purity was 94.1% [IQR, 86.0–97.3] as determined by flow cytometry (FACS Canto II with FACSDiva Software; BD Biosciences, Heidelberg, Germany) using anti-CD14-APC, anti-CD3-PECy7, and anti-CD66-FITC (BD Bioscience, San Jose, CA). See Figure S1 for the gating strategy.

#### *Ex vivo* stimulations

Numbers of freshly isolated neutrophils were adjusted to 5 × 10^6^ cells/mL in Roswell Park Memorial Institute (RPMI) medium supplemented with 10% sterile fetal calf serum (HyClone, South Logan, UT), 200mM glutamax (Thermofisher, Waltham, MA), 100μM pyruvate (ThermoFisher, Waltham, MA), and 50μg/ml gentamycin (Lonza, Basel, Switzerland) in a cell-repellent surface 48-well plate (Greiner Bio-one, Kremsmünster, Austria). Cells were then stimulated for 2 h at 37°C with 5% CO2 and 95% humidity with lipopolysaccharide (LPS; *Escherichia coli* 0111:B4 Ultrapure, 100 ng/mL, Invivogen, Toulouse, France) or heat-killed (30 min at 70°C) *Klebsiella pneumoniae* (ATCC43816; equivalent of 12.5 x 10^6^ CFU/mL). After 2 h neutrophils were centrifuged for 8 min at 1400 revolutions per minute, after which cell supernatants were stored at −80°C until further analysis. MPO release was measured in the stored supernatants using Luminex (R&D, Minneapolis, MN).

#### RNA isolation, library preparation and sequencing

Total RNA was purified using the AllPrep DNA/RNA mini kit following the manufacturer’s instructions (Qiagen, Germany). All samples had high RNA integrity (>9 according to bioanalysis with Agilent). Total RNA and genomic DNA concentrations were determined by Qubit 2.0 Fluorometer (Life Technologies, Carlsbad, CA, USA). RNA-sequencing libraries were prepared from 200ng total RNA using KAPA RNA Hyperprep with RiboErase (Roche, Switzerland) library kits. Libraries were sequenced using the Illumina HiSeq4000 instrument (Illumina, USA) to generate single reads (50bp). The sequencing depth was approximately 40 million reads per sample. Sequencing data were preprocessed and mapped against the Genome Reference Consortium human genome build 38 (GRCh38).^27^^,^^29^

#### Metabolomics

Metabolomic analysis on snapfrozen pellets of 1 x 10^6^ neutrophils was performed with liquid chromatography coupled to mass spectrometry as previously described.^24^^,^^30^ In a 2 mL tube, the following amounts of internal standard dissolved in water were added to each sample of 1 million snapfrozen neutrophils: adenosine-15N5-monophosphate (5 nmol), adenosine-15N5-triphosphate (5 nmol), D4-alanine (0.5 nmol), D7-arginine (0.5 nmol), D3-aspartic acid (0.5 nmol), D3-carnitine (0.5 nmol), D4-citric acid (0.5 nmol), 13C1-citrulline (0.5 nmol), 13C6-fructose-1,6-diphosphate (1 nmol), 13C2-glycine (5 nmol), guanosine-15N5-monophosphate (5 nmol), guanosine-15N5-triphosphate (5 nmol), 13C6-glucose (10 nmol), 13C6-glucose-6-phosphate (1 nmol), D3-glutamic acid (0.5 nmol), D5-glutamine (0.5 nmol), D5-glutathione (1 nmol), 13C6-isoleucine (0.5 nmol), D3-lactic acid (1 nmol), D3-leucine (0.5 nmol), D4-lysine (0.5 nmol), D3-methionine (0.5 nmol), D6-ornithine (0.5 nmol), D5-phenylalanine (0.5 nmol), D7-proline (0.5 nmol), 13C3-pyruvate (0.5 nmol), D3-serine (0.5 nmol), D6-succinic acid (0.5 nmol), D4-thymine (1 nmol), D5-tryptophan (0.5 nmol), D4-tyrosine (0.5 nmol), D8-valine (0.5 nmol). Subsequently, solvents were added to achieve a total volume of 500 μL methanol, 500 μL water and 1 mL chloroform. After thorough mixing, samples were centrifuged for 10 min at 14.000 rpm. The polar top layer was transferred to a new 1.5 mL tube and dried using a vacuum concentrator at 60°C. Dried samples were reconstituted in 100 μL 6:4 (v/v) methanol:water. Metabolites were analyzed using a Waters Acquity ultra-high performance liquid chromatography system coupled to a Bruker Impact II Ultra-High Resolution Qq-Time-Of-Flight mass spectrometer. Samples were kept at 12°C during analysis and 5 μL of each sample was injected. Chromatographic separation was achieved using a Merck Millipore SeQuant ZIC-cHILIC column (PEEK 100 × 2.1 mm, 3 μm particle size). Column temperature was held at 30°C. Mobile phase consisted of (A) 1:9 (v/v) acetonitrile:water and (B) 9:1 (v/v) acetonitrile:water, both containing 5 mmol/L ammonium acetate. Using a flow rate of 0.25 mL/min, the LC gradient consisted of: Dwell at 100% Solvent B, 0–2 min; Ramp to 54% Solvent B at 13.5 min; Ramp to 0% Solvent B at 13.51 min; Dwell at 0% Solvent B, 13.51–19 min; Ramp to 100% B at 19.01 min; Dwell at 100% Solvent B, 19.01–19.5 min. Column was equilibrated by increasing flow rate to 0.4 mL/min at 100% B for 19.5–21 min. MS data were acquired using negative and positive ionization in full scan mode over the range of m/z 50–1200. Data were analyzed using Bruker TASQ software version 2021.1.2.452. All reported metabolite intensities were normalized to internal standards with comparable retention times and response in the MS. Metabolite identification has been based on a combination of accurate mass, (relative) retention times, ion mobility data and fragmentation spectra, compared to the analysis of a library of standards.

### Quantification and statistical analysis

All statistical analyses were performed using R version 4.0.4. Significance was defined as p < 0.05, corrected for multiple testing using the Benjamini-Hochberg method where mentioned. In the box and whisker plots, data are represented with a median line and a box indicating the interquartile range. Correlation analyses were performed using the R-package corrr, and Spearmans’ rho was calculated to quantify the strength of the correlation. Differential gene expression analysis were performed using DESeq2.^31^ Gene set enrichment analysis was performed using the fgsea package and the Reactome pathway database.^15^ Per-sample pathway scores were calculated as follows: first, the expression of every gene was scaled to be in the range of −1 and 1, using a modified logistic activation function. The beta coefficient in this function was the absolute value of the coefficient from a univariate weighted logistic regression, with the gene as predictor, and the sample group as the categorical outcome. The pathway score was then calculated as the mean of the scaled expression values over all the pathway’s constituent genes. The weighted gene co-expression network analysis (WGCNA) is described in detail in the Supplemental Methods. The R-package degPatterns was used to find clusters of genes with similar trends of expression between groups. For the effect size barplots, we calculated Hedges’ g^32^ between patients with CAP at admission and control subjects.

#### Supplemental Methods

##### Weighted gene co-expression network analysis

First, low expressed genes were removed by selecting the 10% most variable genes.^33^ Outlier detection was performed using hierarchical average linkage clustering (Figure S4). To acquire the most appropriate power (β) for a scale-free network, we evaluate the scale independence and mean connectivity using a β value ranging from 1 to 20.^17^ Using this method, we searched for a signed R squared close to 0.9 in the combination with flattening of the mean connectivity curve (Figure S5). These results were compared to the results of the ““pickSoftThreshold” function.^17^ Using this ideal power value, a Pearson’s correlation matrix of all gene pairs was transformed into an adjacency matrix and topological overlap matrix. Modules, which represent genes with high topological overlap, were identified using the dynamic tree cut algorithm with a minimum cluster size of 40 genes.^17^ Modules that were highly similar, correlation coefficient >0.75, were merged into one module (Figure S6). For each gene, the Module Membership (MM) was calculated by correlating its gene expression profile with the module eigengene (ME); the first principal component of a module.^34^ Genes weakly correlated with all modules (|MM|<0.7) were not assigned.^35^ To select the most valuable module, we used two different approaches. First, we compared the ME between CAP patients and healthy controls using a Wilcoxon rank-sum test. Second, we analyzed the correlation of the modules with CAP by evaluating the average absolute gene significance for all genes in a given module. Functional profiling was conducted in g:Profiler using on MM based ordered query.^36^ A BH-adjusted p value <0.05 in the Reactome database was deemed significant.^15^ To visualize a module, we used Cytoscape.^37^ At last, hub genes were identified with the Maximal Clique Centrality algorithm of the Cytospace plugin CytoHubba.^38^

## Data Availability

• All data reported in this paper will be shared by the lead contact upon request. • This paper does not report original code. • Any additional information required to reanalyze the data reported in this paper is available from the lead contact upon request.
