# Supplementary material for: Inflammatory and glycolytic programs underpin a primed blood neutrophil state in patients with pneumonia
Source: iScience. 2023 Jun 19;26(7):107181. doi: 10.1016/j.isci.2023.107181 (PMC10366455; doi:10.1016/j.isci.2023.107181)
Supplement: Document S1. Figures S1–S9 and Tables S1–S4 [file mmc1.pdf]

iScience, Volume *26*

Supplemental information

Inflammatory and glycolytic programs underpin a primed blood neutrophil state in patients with pneumonia

Alex R. Schuurman, Joe M. Butler, Erik H.A. Michels, Natasja A. Otto, Xanthe Brands, Bastiaan W. Haak, Fabrice Uhel, Augustijn M. Klarenbeek, Daniël R. Faber, Bauke V. Schomakers, Michel van Weeghel, Alex F. de Vos, Brendon P. Scicluna, Riekelt H. Houtkooper, W. Joost Wiersinga, and Tom van der Poll

Supplemental information

Figure S1: Gating strategy

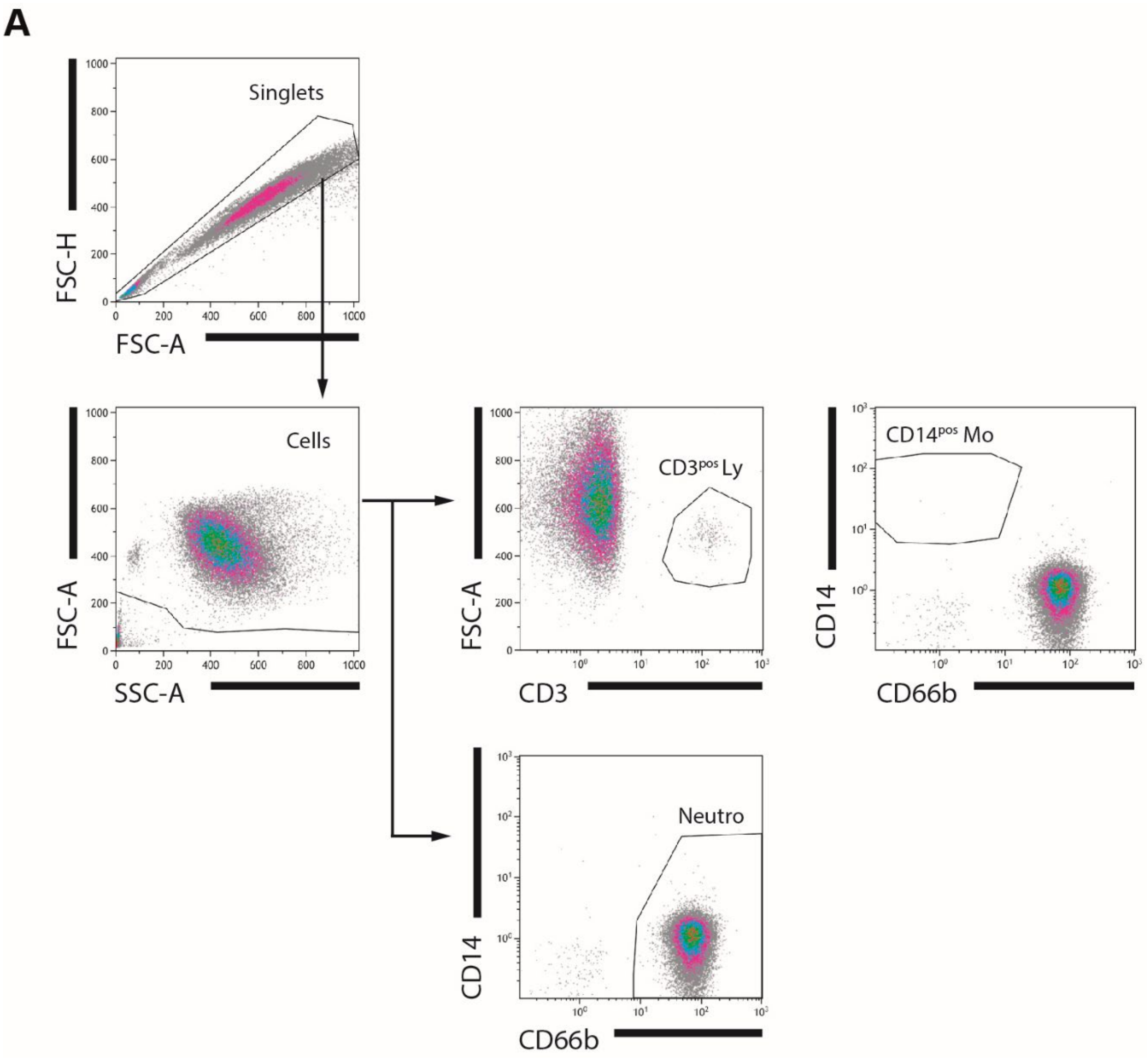

Figure S1: **Gating strategy to ascertain neutrophil purity based on CD3, CD14 and CD66b, related to Figure 1.**

Figure S2: Plasma biomarkers between patients with CAP at admission, and controls

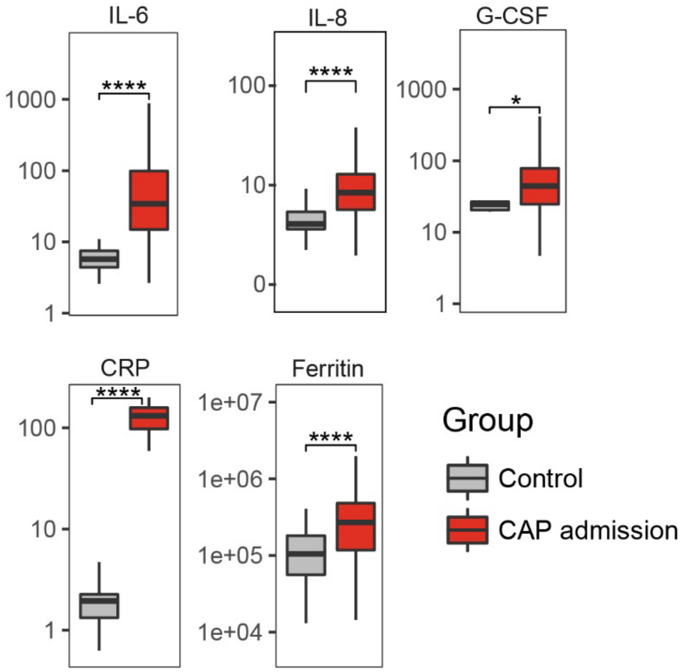

Figure S2. **Boxplots depicting plasma biomarkers relevant for systemic inflammation and neutrophils activation**, **related to Figure 1**. Significance tested with a Wilcoxon rank-sum test. Values are in pg/ml, except for CRP (mg/L). * $p < 0.05$, **** $p < 0.0001$.

Figure S3:

A)

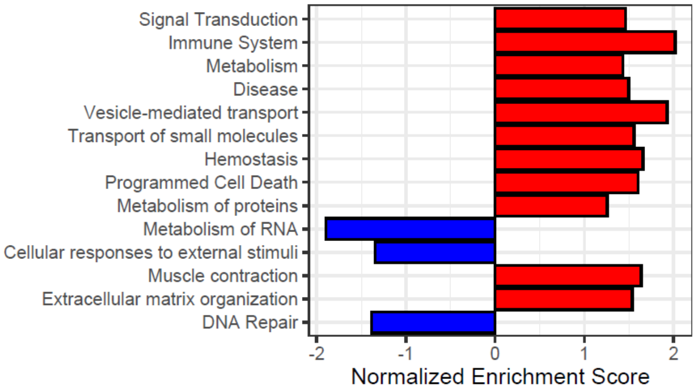
Signal Transduction
Immune System
Metabolism
Disease
Vesicle-mediated transport
Transport of small molecules
Hemostasis
Programmed Cell Death
Metabolism of proteins
Metabolism of RNA
Cellular responses to external stimuli
Muscle contraction
Extracellular matrix organization
DNA Repair
-2
-1
0
1
2
Normalized Enrichment Score

B)

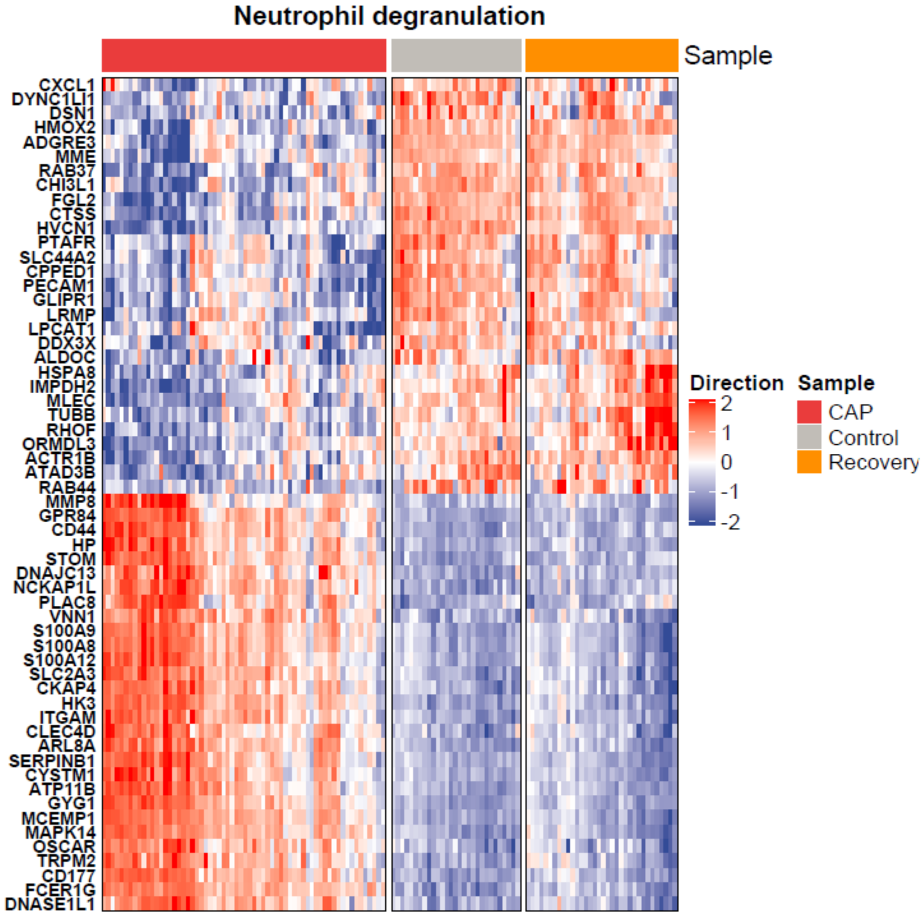
Neutrophil degranulation
Sample
CXCL1
DYNC1LI1
DSN1
HMOX2
ADGRE3
MME
RAB37
CHI3L1
FGL2
CTSS
HVCN1
PTAFR
SLC44A2
CPPED1
PECAM1
GLIPR1
LRMP
LPCAT1
DDX3X
ALDOC
HSPA8
IMPDH2
MLEC
TUBB
RHOF
ORMDL3
ACTR1B
ATAD3B
RAB44
MMP8
GPR84
CD44
HP
STOM
DNAJC13
NCKAP1L
PLAC8
VNN1
S100A9
S100A8
S100A12
SLC2A3
CKAP4
HK3
ITGAM
CLEC4D
ARL8A
SERPINB1
CYSTM1
ATP11B
GYG1
MCEMP1
MAPK14
OSCAR
TRPM2
CD177
FCER1G
DNASE1L1
Direction
2
1
0
-1
-2
Sample
CAP
Control
Recovery

Figure S3: **Transcriptomic analyses CAP admission versus controls, related to Figure 2.** A) All Reactome mother pathways significant after BH-correction for multiple testing. Ordered by significance. B) Heatmap of the top 10% upregulated, and top 10% downregulated significant genes in the neutrophil degranulation pathway between CAP admission and controls.

Figure S4: Hierarchical average linkage clustering to detect outlier samples

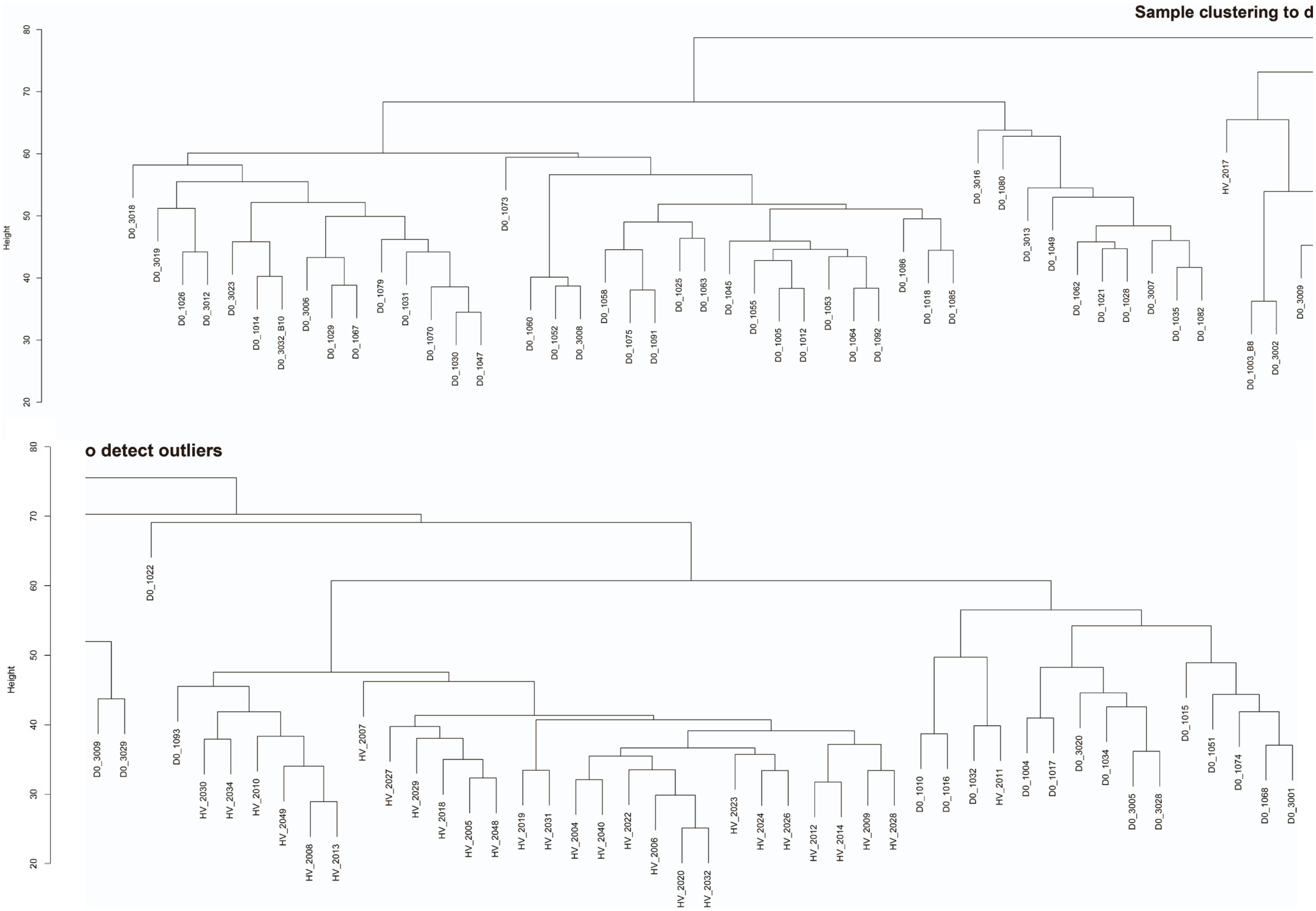

Figure S4: **Hierarchical average linkage clustering, related to Figure 4.** The upper panel resembles the left side and the lower panel resembles the right side of the dendrogram. Samples starting with "HV" are non-infectious controls.

Figure S5:

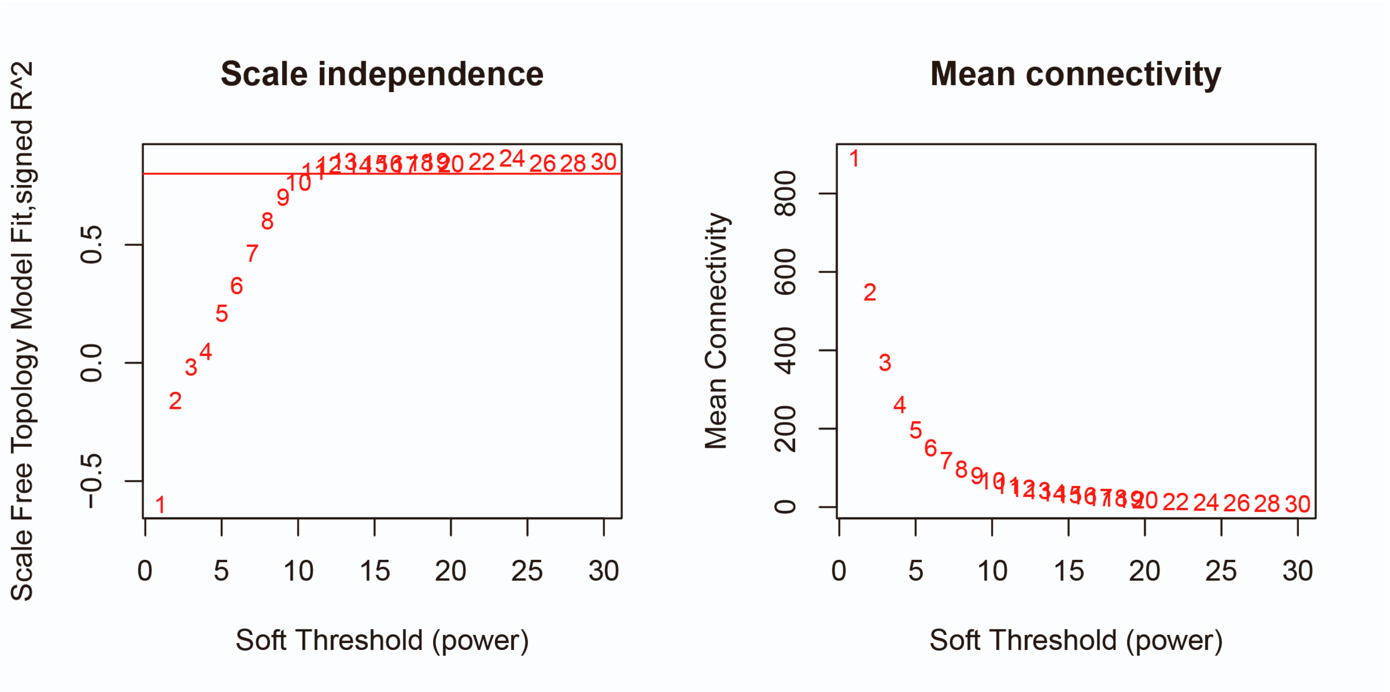

Figure S5: **Weighted gene co-expression network analysis (WGCNA) network construction, related to Figure 4.**

The left panel shows the Scale independence by evaluating the scale-free fit index (signed R^2 for a scale free network) of several Soft Thresholds (powers). The red line indicates a R^2 of 0.8. The right panel shows the Mean connectivity of several powers. A power of 13 showed to be ideal. At this power value, a high R^2 (close to 0.9) in combination of flattening of the mean connectivity curve is observed

Figure S6:

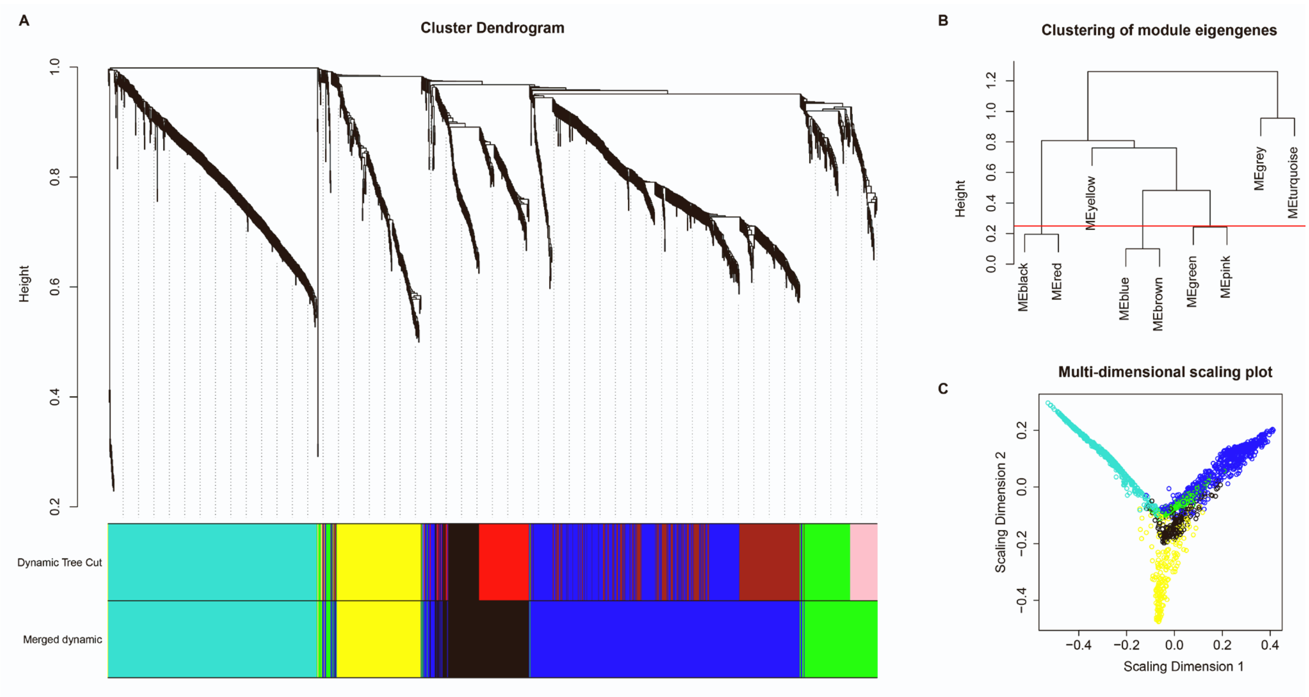

Figure S6: Weighted gene co-expression network analysis cluster dendrogram and cluster assignment, related to Figure 4. A) Cluster dendrogram and assigned modules. Clustering of genes was based on dissimilarity (1 – values of the topological overlap matrix). The minimal cluster size was set to 40 genes. Branches characterize groups of highly correlated genes which are represented by colors referred to as modules. Clustering of 1638 genes of 93 patients resulted in 8 modules, excluding the grey module. The colors within the Dynamic Tree Cut bar plot represent the modules before merging based on similarity. The colors in the Merged dynamic colors barplot represent the modules after merging. B) Clustering of module eigengenes. Modules with a correlation coefficient greater than 0.75 were merged. The black and red module were merged into the black module, the blue and brown module into the brown module, and the green and pink module into the pink module. After merging, 5 modules remained. C) Multi-dimensional scaling plot displaying the expression of genes in the 5 modules in a two dimensional manner. Dots are colored by their corresponding module. Abbreviations: ME; module eigengene.

Figure S7:

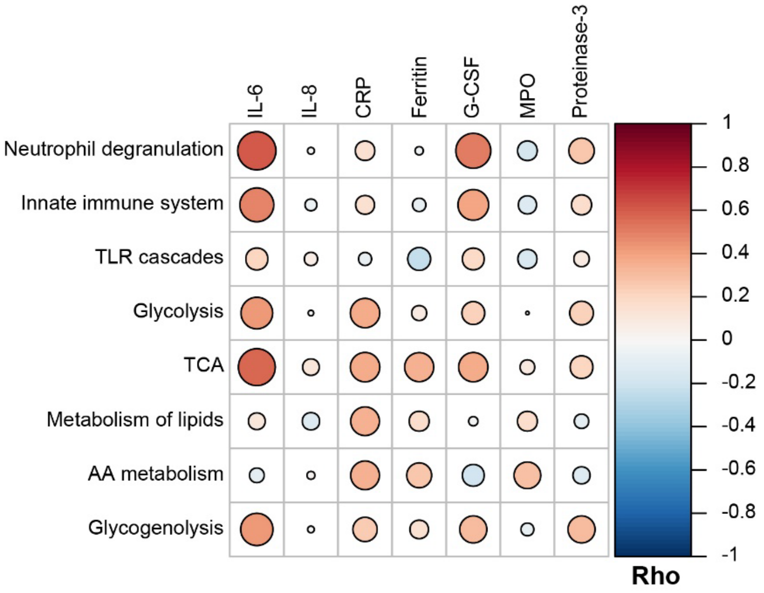

Figure S7: **All correlations between neutrophil transcriptomic pathway scores and plasma biomarkers, related to Figure 4.** Correlation plot depicting correlations between neutrophil transcriptomic pathway scores and plasma biomarkers. The size and the color of the circles are proportional to the strength of Spearman's rho.

Figure S8:

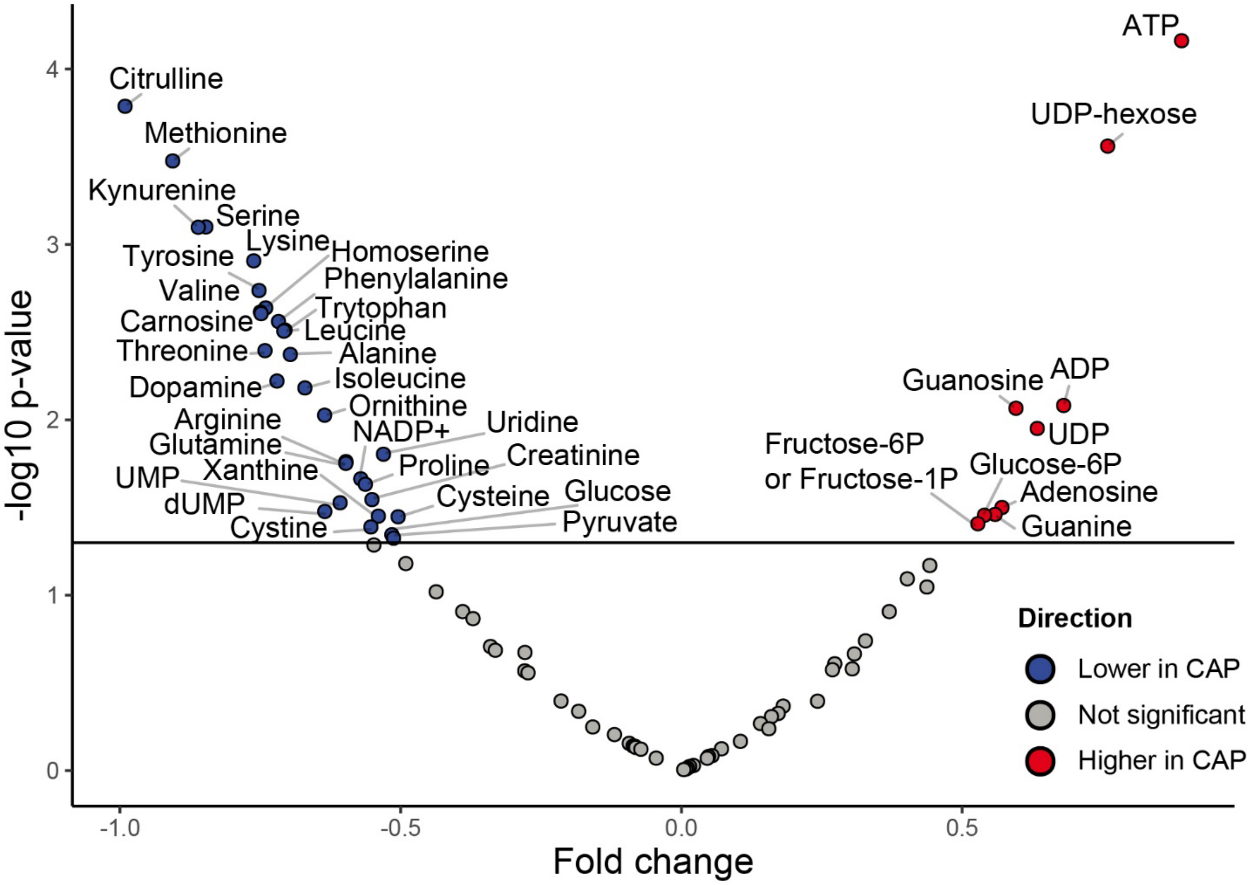

Figure S8: **Volcano plot of all metabolites, related to Figure 5.** Volcano plot depicting the metabolomic differences between neutrophils from patients with CAP at admission, and controls. The x-axis depicts the fold change, the y-axis the unadjusted p-value. Only significant metabolites are annotated.

Figure S9:

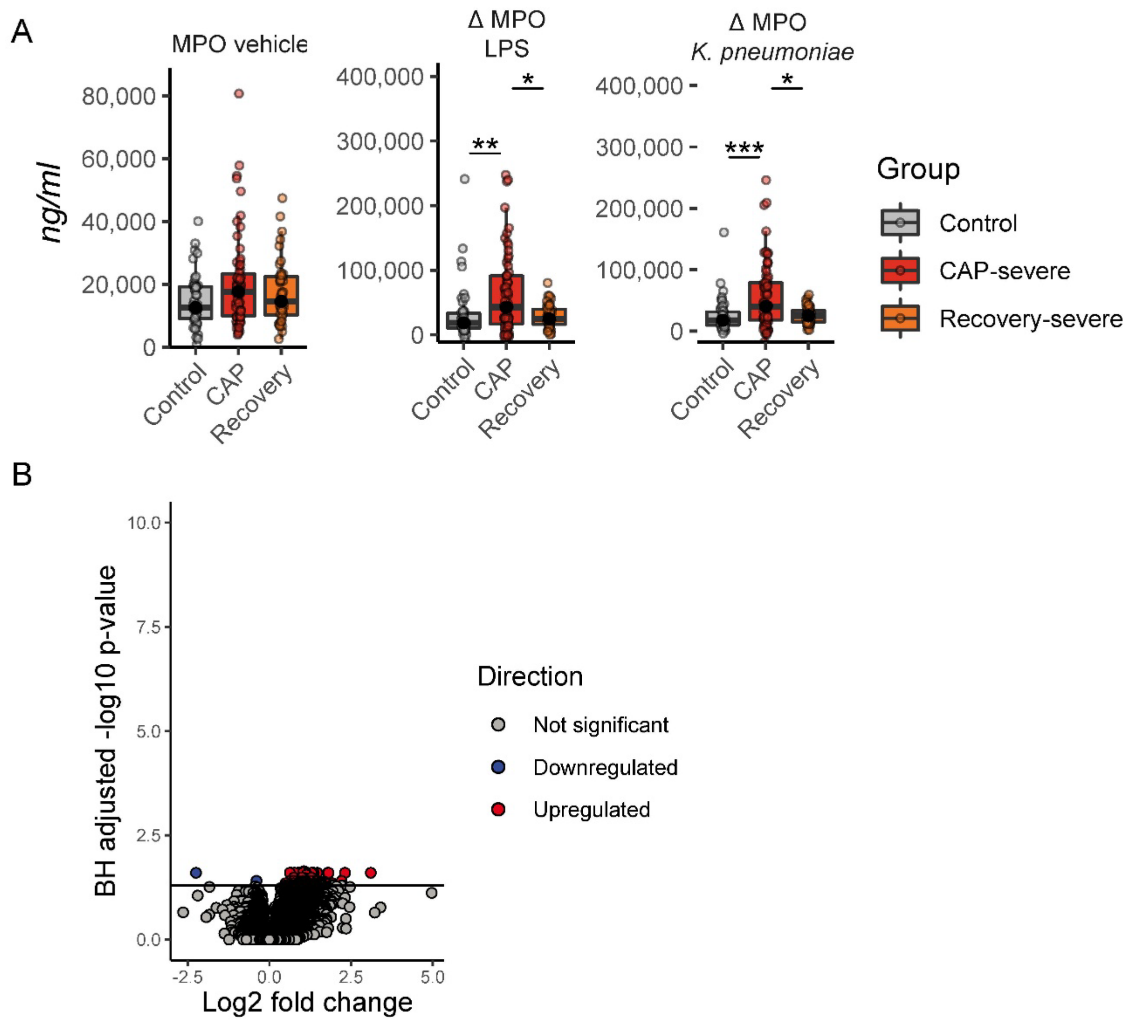

Figure S9: **Sub-analysis of neutrophil profile recovery in the most severely ill patients, related to Figure 1 and 2.**

A) MPO release of neutrophils after ex vivo stimulation (47 control, 66 CAP admission with Pneumonia Severity Index class 4 or higher, 43 CAP recovery with Pneumonia Severity Index class 4 or higher); delta (Δ) values represent the stimulated value (with LPS or K. pneumoniae respectively) minus the unstimulated (vehicle) value. Data are shown as boxplots with individual data points. * p-value < 0.05, ** p-value < 0.01, *** p-value < 0.001 (by Wilcoxon rank-sum test). B) Volcano plot showing the differentially expressed genes (DEGs) in neutrophils from 21 patients with severe CAP (one month follow-up sample), and 29 controls. Each dot represents a gene. The X-axis denotes the log2 fold change between groups, while the Y-axis shows the Benjamini-Hochberg adjusted -log10 p-value.

Table S1: Genes in cluster 3 and 4, related to Figure 3.

Symbol	ENTREZ ID	Log2 fold change	BH-adjusted P	Cluster
LMF1	64788	-1,890	0,008577	3
CCAR1	55749	0,191	0,016413	3
HNRNPM	4670	0,206	0,025735	3
OGDH	4967	0,208	0,006004	3
OGG1	4968	0,211	0,004637	3
PIP4K2A	5305	0,214	0,034592	3
PPP2R5D	5528	0,216	0,029953	3
ITGAL	3683	0,217	0,014645	3
TUT4	23318	0,222	0,029230	3
ARHGDIA	396	0,229	0,011724	3
VTI1B	10490	0,231	0,020323	3
MACO1	55219	0,236	0,024649	3
OPA1	4976	0,248	0,017140	3
TMEM63B	55362	0,250	0,023208	3
ATMIN	23300	0,255	0,039869	3
RSU1	6251	0,266	0,035186	3
EIF4G1	1981	0,270	0,012351	3
SMG6	23293	0,293	0,004803	3
UTP14C	9724	0,304	0,029975	3
EXOC2	55770	0,311	0,039830	3
AEBP2	121536	0,312	0,004449	3
FOXK1	221937	0,315	0,040339	3
ARL17A	51326	0,317	0,611594	3
ZBTB7A	51341	0,338	0,001605	3
PHF19	26147	0,339	0,042646	3
DENND2D	79961	0,350	0,020283	3
FTSJ3	117246	0,352	0,006575	3
CRACR2A	84766	0,358	0,000382	3
CITED4	163732	0,387	0,043668	3
RASAL3	64926	0,408	0,000614	3
BBS7	55212	0,412	0,007995	3
PTPA	5524	0,450	0,006291	3
ZNF444	55311	0,451	0,000289	3
COMMD4	54939	0,462	0,016801	3
PWWP2B	170394	0,477	0,006389	3
SPTBN1	6711	0,499	0,009514	3
FBXO44	93611	0,531	0,045104	3
C19orf12	83636	0,600	0,020516	3
RAB23	51715	0,603	0,038377	3
P2RX4	5025	0,614	0,037488	3
NEO1	4756	0,666	0,018147	3
SLAMF7	57823	0,770	0,023819	3
IL21R	50615	0,783	0,026072	3
NEIL1	79661	0,855	0,006171	3
LGALS3BP	3959	0,888	0,003241	3
GALM	130589	1,019	0,006799	3
SPTB	6710	1,024	0,002564	3
DDX60	55601	1,100	0,010542	3
RSPH4A	345895	1,123	0,026483	3

ARL17A	51326	1,314	0,044447	3
BCYRN1	618	1,391	0,005771	3
E2F1	1869	1,452	0,000529	3
RTP4	64108	1,459	0,007795	3
OAS1	4938	1,484	0,014113	3
IFI44	10561	1,507	0,005862	3
ATP1B2	482	1,542	0,004279	3
EPSTI1	94240	1,545	0,003220	3
TSPAN4	7106	1,571	0,010492	3
RMI2	116028	1,588	0,008329	3
OAF	220323	1,595	0,008213	3
IFI44L	10964	1,681	0,007037	3
KCNC3	3748	1,687	0,009402	3
GBP1P1	400759	1,718	0,008187	3
CDC42EP1	11135	1,809	0,012137	3
LY6E	4061	2,025	0,000629	3
ACHE	43	2,301	0,000717	3
LINC02528	105378020	2,624	0,006088	3
SNORA65	26783	-1,131	0,036180	4
LOC101927914	101927914	-1,077	0,037692	4
LIN37	55957	-0,310	0,029153	4
BACH1	571	-0,250	0,001086	4
FAM131A	131408	-0,223	0,031853	4
USP15	9958	-0,215	0,019730	4
ZBTB18	10472	-0,214	0,009157	4
RYBP	23429	-0,195	0,033080	4
LTN1	26046	-0,192	0,000294	4
VPS4B	9525	-0,175	0,008621	4
SLC35F5	80255	-0,174	0,039130	4
AKIRIN1	79647	-0,164	0,028477	4
CHMP3	51652	-0,161	0,016955	4
PDCD6	10016	-0,122	0,032608	4
STAP1	26228	1,448	0,007038	4

**Table S2: Significant Reactome child pathways under 'immune system' Pathway, related to Figure 3.**	**BH-adjusted P**	**ID**
Neutrophil degranulation	0,010444	R-HSA-6798695
Innate Immune System	0,010444	R-HSA-168249
Toll-like Receptor Cascades	0,010444	R-HSA-168898
Growth hormone receptor signaling	0,011618	R-HSA-982772
Regulation of TLR by endogenous ligand	0,015896	R-HSA-5686938
Toll Like Receptor 4 (TLR4) Cascade	0,010444	R-HSA-166016
Interleukin-1 family signaling	0,010444	R-HSA-446652
TRAF6 mediated NF-kB activation	0,041639	R-HSA-933542
Regulation of actin dynamics for phagocytic cup formation	0,016835	R-HSA-2029482
Toll Like Receptor 2 (TLR2) Cascade	0,015893	R-HSA-181438
Interleukin-4 and Interleukin-13 signaling	0,012265	R-HSA-6785807
Antigen processing-Cross presentation	0,014154	R-HSA-1236975
Signaling by Interleukins	0,010444	R-HSA-449147
Fcgamma receptor (FCGR) dependent phagocytosis	0,018985	R-HSA-2029480
Class I MHC mediated antigen processing & presentation	0,010444	R-HSA-983169
Cytokine Signaling in Immune system	0,010444	R-HSA-1280215
MHC class II antigen presentation	0,020829	R-HSA-2132295
Interferon gamma signaling	0,027392	R-HSA-877300
Toll Like Receptor 7/8 (TLR7/8) Cascade	0,033063	R-HSA-168181
Toll Like Receptor 9 (TLR9) Cascade	0,030062	R-HSA-168138
Adaptive Immune System	0,010444	R-HSA-1280218
Antigen processing: Ubiquitination & Proteasome degradation	0,014396	R-HSA-983168
Interferon Signaling	0,049523	R-HSA-913531

Table S3: All differential genes in the neutrophil degranulation pathway, related to Figure 3.

Symbol	ENTREZ ID	Log2 fold change	P value	BH-adjusted P
FCER1G	2207	2,6999	1,68E-89	1,83E-85
CD177	57126	7,2683	4,07E-87	2,96E-83
MCEMP1	199675	4,5599	1,42E-81	6,19E-78
GYG1	2992	3,6716	5,50E-73	1,71E-69
GPR84	53831	4,6447	1,29E-71	3,13E-68
HK3	3101	3,0169	1,42E-61	1,81E-58
STOM	2040	3,0032	5,23E-58	4,95E-55
MAPK14	1432	1,8589	4,67E-56	3,91E-53
CD44	960	2,3304	1,32E-54	9,57E-52
HP	3240	4,6022	1,87E-49	8,68E-47
NCKAP1L	3071	1,2208	4,87E-47	1,80E-44
TRPM2	7226	3,1228	9,75E-45	2,95E-42
ARL8A	127829	1,0951	1,66E-44	4,96E-42
VNN1	8876	3,2740	6,34E-44	1,84E-41
S100A9	6280	1,6312	7,44E-42	1,89E-39
HVCN1	84329	-2,1993	1,86E-41	4,56E-39
ATP11B	23200	1,6817	7,75E-41	1,80E-38
S100A8	6279	1,9959	1,19E-40	2,70E-38
CYSTM1	84418	2,6092	1,62E-39	3,40E-37
S100A12	6283	3,0784	2,55E-38	5,10E-36
SLC2A3	6515	1,5505	5,83E-38	1,13E-35
DNAJC13	23317	1,3635	5,91E-38	1,13E-35
PLAC8	51316	3,6601	3,91E-37	6,89E-35
MMP8	4317	5,0044	9,26E-37	1,58E-34
SERPINB1	1992	1,7320	1,16E-36	1,95E-34
DNASE1L1	1774	1,2339	1,97E-36	3,26E-34
ITGAM	3684	1,4965	5,33E-36	8,66E-34
CLEC4D	338339	2,4747	1,84E-35	2,82E-33
CKAP4	10970	1,7007	6,29E-34	8,56E-32
OSCAR	126014	1,2955	1,38E-33	1,87E-31
CR1	1378	1,7641	3,11E-33	4,02E-31
CD59	966	1,6891	5,07E-33	6,50E-31
ARG1	383	3,0777	3,45E-32	4,13E-30
C3AR1	719	2,7888	3,70E-31	4,12E-29
FCAR	2204	1,6355	7,54E-31	8,13E-29
CLEC5A	23601	2,7806	4,15E-30	4,29E-28
NRAS	4893	1,1637	1,07E-28	1,00E-26
GNS	2799	1,1633	5,67E-28	5,00E-26
MMP9	4318	2,4693	3,01E-27	2,48E-25
S100A11	6282	0,9536	2,39E-26	1,85E-24
LAIR1	3903	2,8599	1,66E-25	1,18E-23
PKM	5315	1,6461	3,50E-25	2,41E-23
NFKB1	4790	0,7680	1,11E-24	7,42E-23
RETN	56729	4,5423	1,61E-24	1,05E-22
ARMC8	25852	0,5558	2,36E-24	1,53E-22
NHLRC3	387921	1,9363	2,94E-24	1,89E-22
ADGRG3	222487	1,1940	5,82E-24	3,65E-22
CTSS	1520	-1,0132	1,88E-23	1,13E-21
DOK3	79930	0,9819	3,42E-23	2,01E-21

QSOX1	5768	1,4157	4,74E-23	2,73E-21
ABCA13	154664	1,6088	2,46E-22	1,34E-20
TSPAN14	81619	0,6763	6,56E-22	3,43E-20
PRDX6	9588	0,9352	9,95E-22	5,09E-20
PRKCD	5580	0,7617	1,30E-21	6,60E-20
PYGL	5836	1,2048	6,08E-21	2,88E-19
NCSTN	23385	0,6297	4,14E-20	1,80E-18
TOM1	10043	0,7142	5,93E-20	2,53E-18
CAPN1	823	0,8268	6,85E-20	2,89E-18
FGR	2268	0,9561	1,14E-19	4,74E-18
GRN	2896	0,9187	1,49E-19	6,09E-18
CD63	967	1,0848	1,84E-19	7,40E-18
RAB27A	5873	0,8810	5,13E-19	1,98E-17
CD53	963	0,5405	5,81E-19	2,23E-17
AGPAT2	10555	0,7476	6,09E-19	2,33E-17
GLIPR1	11010	-0,9080	1,96E-18	7,15E-17
MGAM	8972	1,3606	3,07E-18	1,10E-16
ATP8B4	79895	1,0820	8,53E-18	2,92E-16
CTSD	1509	1,0438	1,14E-17	3,86E-16
MGST1	4257	2,2483	2,30E-17	7,51E-16
ALOX5	240	1,0582	3,92E-17	1,25E-15
SERPINA1	5265	0,8185	5,01E-17	1,57E-15
ACAA1	30	0,7543	7,87E-17	2,41E-15
LTA4H	4048	1,5155	1,06E-16	3,19E-15
CPPED1	55313	-0,8625	1,16E-16	3,48E-15
RAB31	11031	0,7473	3,14E-16	8,99E-15
DERA	51071	0,9721	3,51E-16	1,00E-14
RAB3A	5864	1,2691	5,68E-16	1,58E-14
RAB10	10890	0,6572	5,68E-16	1,58E-14
HSPA1B	3304	1,1282	2,64E-15	6,86E-14
RAB37	326624	-0,8258	2,70E-15	7,00E-14
HMOX2	3163	-0,7137	3,38E-15	8,65E-14
DYNC1LI1	51143	-0,5755	6,63E-15	1,65E-13
SELL	6402	0,7742	9,88E-15	2,41E-13
DSN1	79980	-0,8792	1,09E-14	2,64E-13
ADGRE3	84658	-1,2823	1,13E-14	2,73E-13
DNAJC5	80331	0,6705	2,03E-14	4,77E-13
LRG1	116844	0,9036	2,40E-14	5,58E-13
LILRB3	11025	0,7415	3,17E-14	7,21E-13
PECAM1	5175	-0,8046	3,75E-14	8,46E-13
FGL2	10875	-1,3795	4,73E-14	1,06E-12
BST1	683	0,8096	7,47E-14	1,63E-12
VAT1	10493	1,1465	7,76E-14	1,69E-12
ACTR1B	10120	-0,6533	9,90E-14	2,13E-12
CYB5R3	1727	0,8001	1,45E-13	3,05E-12
CYBA	1535	0,5299	1,77E-13	3,67E-12
PGM1	5236	0,8254	2,40E-13	4,91E-12
VAPA	9218	0,8011	2,60E-13	5,29E-12
LAMP1	3916	0,5869	3,58E-13	7,17E-12
SIGLEC9	27180	0,8677	4,51E-13	8,89E-12
RAB44	401258	-2,3795	9,40E-13	1,77E-11

ALDH3B1	221	0,8544	9,85E-13	1,85E-11
CHI3L1	1116	-2,3095	1,02E-12	1,92E-11
RHOG	391	0,6095	1,06E-12	1,97E-11
CTSA	5476	0,8596	1,68E-12	3,08E-11
CEACAM1	634	1,5259	1,80E-12	3,29E-11
BRI3	25798	0,5919	1,84E-12	3,36E-11
GM2A	2760	1,6642	1,84E-12	3,36E-11
TIMP2	7077	0,5767	1,93E-12	3,51E-11
CAB39	51719	0,6989	2,35E-12	4,23E-11
CAP1	10487	0,5504	5,11E-12	8,85E-11
TLR2	7097	0,7199	6,01E-12	1,03E-10
SIGLEC5	8778	0,8482	6,46E-12	1,10E-10
RHOF	54509	-0,6621	1,20E-11	1,97E-10
COPB1	1315	0,4750	1,47E-11	2,39E-10
CD55	1604	0,9399	1,85E-11	2,97E-10
PTAFR	5724	-0,5403	2,91E-11	4,58E-10
GCA	25801	0,8305	9,17E-11	1,35E-09
CD58	965	0,5223	1,19E-10	1,73E-09
AMPD3	272	0,7530	1,43E-10	2,04E-09
TRAPPC1	58485	0,5042	2,63E-10	3,61E-09
OLFM4	10562	2,8703	2,79E-10	3,81E-09
CTSH	1512	1,3996	2,91E-10	3,97E-09
PSMD1	5707	0,6932	3,30E-10	4,47E-09
CPNE3	8895	0,6420	4,84E-10	6,42E-09
IMPDH2	3615	-0,9137	9,95E-10	1,27E-08
LCN2	3934	2,2916	2,24E-09	2,72E-08
LRMP	4033	-0,5238	2,61E-09	3,13E-08
MLEC	9761	-1,0341	3,05E-09	3,63E-08
VPS35L	57020	0,6399	3,05E-09	3,63E-08
OSTF1	26578	0,3737	6,43E-09	7,31E-08
RAP2B	5912	0,6360	9,60E-09	1,07E-07
NBEAL2	23218	0,7277	9,84E-09	1,10E-07
MMP25	64386	0,6795	9,87E-09	1,10E-07
ATAD3B	83858	-0,8779	1,05E-08	1,16E-07
AGA	175	1,0681	1,59E-08	1,72E-07
MME	4311	-1,0340	4,16E-08	4,27E-07
TUBB	203068	-0,5230	5,16E-08	5,20E-07
PGLYRP1	8993	0,9533	5,96E-08	5,96E-07
RAB7A	7879	0,3892	7,94E-08	7,82E-07
IDH1	3417	0,6200	9,52E-08	9,27E-07
SLC11A1	6556	0,8590	9,89E-08	9,61E-07
GMFG	9535	0,4022	1,08E-07	1,04E-06
PRCP	5547	0,4561	1,49E-07	1,41E-06
CNN2	1265	0,4617	1,54E-07	1,45E-06
ITGB2	3689	0,5590	2,06E-07	1,91E-06
TOLLIP	54472	0,4204	2,22E-07	2,05E-06
FCGR2A	2212	0,3782	2,96E-07	2,68E-06
RAB6A	5870	0,3502	3,25E-07	2,91E-06
FPR1	2357	0,4159	3,48E-07	3,11E-06
ORMDL3	94103	-0,6259	3,86E-07	3,42E-06
BPI	671	1,2381	4,36E-07	3,84E-06

SLC44A2	57153	-0,4234	4,74E-07	4,14E-06
RAP2C	57826	0,3837	5,01E-07	4,35E-06
PGM2	55276	0,5854	5,42E-07	4,70E-06
LGALS3	3958	0,8488	6,91E-07	5,88E-06
CD14	929	0,7277	7,32E-07	6,20E-06
CYBB	1536	1,0005	7,53E-07	6,37E-06
PSMD6	9861	0,3378	7,59E-07	6,41E-06
DDX3X	1654	-0,3746	7,71E-07	6,51E-06
HSPA8	3312	-0,6836	7,83E-07	6,60E-06
GUSB	2990	0,6256	8,66E-07	7,23E-06
CSTB	1476	0,4803	9,67E-07	8,02E-06
ROCK1	6093	0,4540	1,05E-06	8,66E-06
PADI2	11240	0,7857	1,10E-06	9,08E-06
LPCAT1	79888	-0,4415	1,32E-06	1,07E-05
ATG7	10533	0,3631	1,39E-06	1,13E-05
NDUFC2	4718	0,8521	1,54E-06	1,23E-05
CPNE1	8904	0,5136	2,06E-06	1,61E-05
AP1M1	8907	0,3183	2,42E-06	1,87E-05
ENPP4	22875	0,9134	3,25E-06	2,46E-05
PSMD12	5718	0,3484	3,45E-06	2,60E-05
HPSE	10855	0,8704	3,97E-06	2,95E-05
ALDOC	230	-0,6967	6,47E-06	4,61E-05
LAMTOR2	28956	0,4169	9,17E-06	6,37E-05
ACTR10	55860	0,3678	9,54E-06	6,60E-05
PLD1	5337	0,3913	9,66E-06	6,67E-05
CXCL1	2919	-0,8340	9,71E-06	6,71E-05
CTSB	1508	0,6368	1,32E-05	8,88E-05
PSMC2	5701	0,6150	1,45E-05	9,69E-05
DYNLT1	6993	0,5226	1,46E-05	9,77E-05
RAB14	51552	0,1986	1,68E-05	0,000111237
ITGAX	3687	0,5471	1,99E-05	0,000129324
FUCA1	2517	-0,7029	2,03E-05	0,000131802
PLEKHO2	80301	0,2954	2,11E-05	0,000136635
HSPA1A	3303	0,5612	2,22E-05	0,000143087
NAPRT	93100	0,5877	2,25E-05	0,000144435
ALDOA	226	0,3756	2,49E-05	0,000158568
GDI2	2665	0,3029	2,58E-05	0,000163497
TMC6	11322	-0,3693	2,68E-05	0,000169355
DIAPH1	1729	0,3326	2,89E-05	0,000181809
SNAP29	9342	0,2100	3,03E-05	0,000189612
ATP11A	23250	0,4109	3,29E-05	0,000203758
SIRPA	140885	0,4141	3,99E-05	0,000243993
PTPN6	5777	0,2544	4,82E-05	0,000289614
PGRMC1	10857	0,6631	5,82E-05	0,000342974
ARSA	410	-0,3796	6,30E-05	0,000368673
AZU1	566	-0,9802	6,46E-05	0,000377693
CRACR2A	84766	0,3581	6,67E-05	0,000388337
PSMB1	5689	-0,2504	6,72E-05	0,000390793
RAB4B	53916	0,2677	6,98E-05	0,000404547
IQGAP1	8826	0,3941	7,41E-05	0,000427325
DEFA4	1669	-1,3301	7,84E-05	0,000449163

MNDA	4332	-0,3490	8,96E-05	0,000507442
LAMP2	3920	-0,2569	9,03E-05	0,000510728
PGAM1	5223	0,3956	0,00010252	0,000573077
RNASE3	6037	-1,3923	0,000106713	0,000594382
CTSG	1511	-1,1751	0,000113107	0,000626632
SLCO4C1	353189	0,5441	0,000114699	0,000634803
ARPC5	10092	0,3390	0,000123448	0,000679605
SVIP	258010	0,5500	0,000133698	0,000731145
ALAD	210	0,4429	0,000135075	0,000737833
GHDC	84514	0,4728	0,000143072	0,000775879
LILRB2	10288	0,4210	0,000143167	0,000776202
GLA	2717	0,3774	0,000152203	0,000818069
P2RX1	5023	0,5523	0,000153692	0,000824241
IGF2R	3482	-0,4283	0,000160797	0,000857702
YPEL5	51646	-0,2846	0,000205503	0,001068451
TCN1	6947	0,7757	0,000229775	0,001179175
GOLGA7	51125	0,3408	0,000244123	0,00124489
CANT1	124583	-0,2426	0,000263818	0,001332849
MS4A3	932	-0,9237	0,000271781	0,001369271
IMPDH1	3614	0,4176	0,000301148	0,001504703
CFD	1675	-1,3966	0,00031422	0,001564283
CCT2	10576	-0,6259	0,000343126	0,001694251
EEF1A1	1915	-0,6511	0,000361087	0,001773297
VCL	7414	0,3758	0,000400559	0,00194566
SDCBP	6386	0,2888	0,000435988	0,002101826
XRCC6	2547	-0,3498	0,000456274	0,002188488
SLPI	6590	0,7085	0,000469285	0,002244471
SERPINB6	5269	0,4652	0,000477531	0,002278909
SRP14	6727	0,2410	0,000490504	0,002335144
STING1	340061	0,6577	0,000529367	0,002499469
STBD1	8987	1,2956	0,000638444	0,002954327
LAMTOR1	55004	0,3045	0,000647406	0,002991354
NIT2	56954	-0,7912	0,00064985	0,003000738
PPIA	5478	-0,5813	0,000782881	0,003548856
PTX3	5806	0,8363	0,000787256	0,00356646
KCMF1	56888	0,2099	0,000788525	0,003570725
HBB	3043	-1,0579	0,000813764	0,003676616
JUP	3728	1,4494	0,000823342	0,003717561
DEGS1	8560	0,3105	0,000929735	0,004144729
DYNLL1	8655	0,4715	0,000939067	0,004183764
C3	718	-0,9271	0,000939794	0,004186144
SERPINB10	5273	1,1204	0,000955859	0,004246421
HEBP2	23593	0,3254	0,00095981	0,004263106
BIN2	51411	0,2642	0,001010869	0,004457323
ATP6AP2	10159	-0,2996	0,001017329	0,004475714
C6orf120	387263	0,3178	0,001051117	0,004601145
GSDMD	79792	0,4283	0,001068874	0,004661084
PNP	4860	0,6917	0,001153138	0,004982682
ERP44	23071	0,1948	0,001178302	0,005080334
CD68	968	0,7122	0,001248096	0,00535056
TBC1D10C	374403	-0,2435	0,001277278	0,005457416

FAF2	23197	0,1762	0,001376715	0,005831972
DGAT1	8694	-0,3075	0,00144495	0,006091429
ANO6	196527	0,5868	0,001725851	0,007097153
UNC13D	201294	0,2664	0,001851243	0,007544385
PTGES2	80142	-0,6693	0,001859625	0,007570139
TMEM63A	9725	-0,2739	0,001894442	0,007694625
PSMD11	5717	0,2397	0,001990499	0,008045794
OLR1	4973	-0,9360	0,002092919	0,008400582
APRT	353	-0,7627	0,00211648	0,008484217
LTF	4057	1,0671	0,002206937	0,008804723
ATP8A1	10396	0,2296	0,002240742	0,008921622
EEF2	1938	-0,5480	0,002266763	0,009016991
RHOA	387	0,2554	0,002272892	0,00903313
PSMD2	5708	0,2149	0,002501452	0,009814429
FOLR3	2352	1,0022	0,002635044	0,010272006
PPIE	10450	-0,7760	0,002757385	0,010682047
FCN1	2219	0,5621	0,002817781	0,010881206
PTPRJ	5795	0,2845	0,002927548	0,011243331
CEP290	80184	-0,5509	0,003122579	0,011895928
MVP	9961	0,2448	0,003439948	0,012966737
KCNAB2	8514	0,2897	0,004122399	0,015175191
C1orf35	79169	-0,3855	0,004139042	0,015227958
ITGAL	3683	0,2173	0,004218191	0,015493003
RAP1B	5908	0,1711	0,00422271	0,015506986
FPR2	2358	0,2792	0,004432432	0,016176298
DPP7	29952	-0,7032	0,004463915	0,016266682
TNFRSF1B	7133	-0,2281	0,005028185	0,018063177
CD33	945	-0,6616	0,005042148	0,018110351
B2M	567	-0,2508	0,005125181	0,018372256
AP2A2	161	0,2166	0,005767528	0,020314039
CDA	978	0,3378	0,006369418	0,022158255
PA2G4	5036	-0,5403	0,006392361	0,022225164
FABP5	2171	1,1390	0,006525175	0,022620676
BST2	684	0,7221	0,006535555	0,022653057
ARHGAP45	23526	-0,2057	0,007454901	0,025318672
CRISP3	10321	0,6518	0,008150771	0,027325959
TYROBP	7305	0,1914	0,008820136	0,029250589
NFASC	23114	1,0430	0,010101418	0,032849956
IQGAP2	10788	0,3609	0,011579056	0,037020028
QPCT	25797	0,3400	0,012056603	0,038299614
SIRPB1	10326	0,2449	0,013135538	0,041269865
CTSZ	1522	0,2024	0,013270077	0,041629626
CTSC	1075	0,3677	0,013545787	0,042351231
ORM1	5004	0,7176	0,014236177	0,044117371
CD36	948	0,9481	0,016514045	0,050135619
ACTR2	10097	0,2340	0,017243268	0,051987415

Table S4: All differential genes between severe CAP recovery and control samples

Symbol	ENTREZ ID	Log2 fold change	P value	BH-adjusted P	Direction
VGLL4	9686	1,061412267	1,01E-06	0,015146297	Up
CSNK2A2	1459	0,87969711	2,90E-06	0,016622172	Up
C4BPA	722	-2,244967662	3,70E-06	0,016622172	Down
UTP3	57050	1,348123448	6,10E-06	0,016622172	Up
CHI3L2	1117	2,314872168	6,95E-06	0,016622172	Up
RAP1GDS1	5910	1,108061605	8,12E-06	0,016622172	Up
AUTS2	26053	1,787526696	8,68E-06	0,016622172	Up
HIRIP3	8479	1,279780222	1,03E-05	0,016622172	Up
MTA2	9219	0,672530064	1,05E-05	0,016622172	Up
CD7	924	1,463995878	1,38E-05	0,016622172	Up
DROSHA	29102	0,946240987	1,41E-05	0,016622172	Up
RPTOR	57521	1,358444238	1,49E-05	0,016622172	Up
TTC37	9652	1,322192673	1,62E-05	0,016622172	Up
EZR	7430	0,770238439	1,65E-05	0,016622172	Up
ATP1A3	478	1,811144063	1,95E-05	0,016622172	Up
PPDPF	79144	1,287700704	2,07E-05	0,016622172	Up
OSTC	58505	1,12203869	2,15E-05	0,016622172	Up
AP3B2	8120	3,104866096	2,17E-05	0,016622172	Up
NUCKS1	64710	1,029435519	2,21E-05	0,016622172	Up
MRPS5	64969	0,628547222	2,22E-05	0,016622172	Up
NDUFA8	4702	1,137561269	2,58E-05	0,018380711	Up
WDSUB1	151525	1,047132702	3,17E-05	0,021584587	Up
USP28	57646	1,288980761	3,47E-05	0,021727244	Up
WWP1	11059	0,748177206	3,58E-05	0,021727244	Up
TMEM209	84928	0,997997885	3,63E-05	0,021727244	Up
PHF14	9678	1,132822786	4,53E-05	0,025907443	Up
FBXW4	6468	0,79499206	4,93E-05	0,025907443	Up
PHF19	26147	0,864287443	5,23E-05	0,025907443	Up
ARID5B	84159	1,131291472	5,38E-05	0,025907443	Up
VEGFB	7423	1,355495929	5,56E-05	0,025907443	Up
TCAF1	9747	1,142215197	5,63E-05	0,025907443	Up
WDR6	11180	0,705958129	5,70E-05	0,025907443	Up
DMWD	1762	1,047065159	5,84E-05	0,025907443	Up
PRKCH	5583	1,39309046	5,91E-05	0,025907443	Up
GABPB2	126626	0,717357127	6,42E-05	0,025907443	Up
SCP2	6342	0,877605422	6,49E-05	0,025907443	Up
CASP9	842	-0,394795676	6,66E-05	0,025907443	Down
CBLB	868	1,027696119	6,78E-05	0,025907443	Up
ZC3H7B	23264	1,234582669	6,78E-05	0,025907443	Up
ZFPM1	161882	0,630202416	6,92E-05	0,025907443	Up
EIF3F	8665	0,740682577	7,30E-05	0,02601848	Up
MRPS30	10884	1,105067365	7,32E-05	0,02601848	Up
ADGRG1	9289	2,213839186	7,48E-05	0,02601848	Up
FOXP4	116113	1,479400687	7,75E-05	0,026092771	Up
SND1	27044	1,008766286	7,85E-05	0,026092771	Up
SSBP3	23648	1,050557678	8,14E-05	0,026469486	Up
SPN	6693	1,654109352	8,57E-05	0,027294275	Up
ZFP90	146198	1,086445171	9,01E-05	0,027605313	Up

CEP78	84131	1,571706169	9,04E-05	0,027605313	Up
ATXN10	25814	1,055406361	9,42E-05	0,028201247	Up
CPLANE1	65250	1,143261261	9,81E-05	0,028801725	Up
HIVEP3	59269	1,82811484	0,000100362	0,028886743	Up
USP11	8237	0,847119324	0,000106567	0,029135892	Up
AEBP2	121536	0,531180003	0,000109029	0,029135892	Up
MFSD6	54842	1,526772911	0,000109722	0,029135892	Up
ANXA6	309	1,204754001	0,000110961	0,029135892	Up
TCAF2	285966	0,904088815	0,000116032	0,029225913	Up
CDR2	1039	0,902619244	0,000116505	0,029225913	Up
ESD	2098	0,810950077	0,000120088	0,029225913	Up
KRR1	11103	0,826468592	0,000122879	0,029225913	Up
MAP4	4134	1,172664901	0,000124759	0,029225913	Up
ELK4	2005	0,927654952	0,000125883	0,029225913	Up
ATIC	471	1,731959693	0,000129644	0,029225913	Up
DCAF1	9730	0,74794276	0,000133819	0,029225913	Up
TMCO4	255104	1,470986717	0,000134481	0,029225913	Up
ABI3	51225	1,670955551	0,000134686	0,029225913	Up
CROT	54677	0,877418292	0,000137497	0,029225913	Up
PARP1	142	1,005393036	0,000138372	0,029225913	Up
MPHOSPH9	10198	0,941477167	0,000139192	0,029225913	Up
ARMH3	79591	0,494074609	0,000140412	0,029225913	Up
CCL5	6352	1,93093204	0,000141962	0,029225913	Up
TOMM7	54543	0,739762968	0,000142546	0,029225913	Up
RIOX1	79697	0,960671101	0,000148164	0,029873116	Up
LIMA1	51474	1,620266124	0,000149695	0,029873116	Up
RAB11FIP5	26056	1,404859025	0,000153247	0,030039479	Up
NKG7	4818	1,738792656	0,000154543	0,030039479	Up
RAP2A	5911	0,919808515	0,000158111	0,030078685	Up
PRNP	5621	1,075622879	0,000158764	0,030078685	Up
RFX7	64864	1,047940659	0,000163591	0,03060578	Up
NNT	23530	0,847382432	0,000167916	0,030904946	Up
TSHZ1	10194	1,243536566	0,00016932	0,030904946	Up
TOX	9760	1,302233572	0,000175426	0,031469354	Up
NBAS	51594	0,909442817	0,000176617	0,031469354	Up
STK39	27347	0,849340081	0,000188915	0,032349684	Up
SLC35B4	84912	1,544795174	0,00018902	0,032349684	Up
ETS1	2113	1,114439881	0,000189985	0,032349684	Up
GDPGP1	390637	0,992853761	0,000190203	0,032349684	Up
CKAP5	9793	0,951980119	0,000193874	0,032603433	Up
ZNF428	126299	1,200756922	0,00020076	0,032614924	Up
KRI1	65095	0,797039111	0,000201271	0,032614924	Up
NIPAL3	57185	0,998657973	0,000204063	0,032614924	Up
HCFC1	3054	1,101727374	0,000204253	0,032614924	Up
BLMH	642	1,392275523	0,000205182	0,032614924	Up
HCST	10870	0,863839243	0,000207225	0,032614924	Up
TCF12	6938	0,757580242	0,000209196	0,032614924	Up
